# Highly Reversible Zinc Metal Anode in a Dilute Aqueous Electrolyte Enabled by a pH Buffer Additive

**DOI:** 10.1002/anie.202212695

**Published:** 2022-12-07

**Authors:** Wei Zhang, Yuhang Dai, Ruwei Chen, Zhenming Xu, Jianwei Li, Wei Zong, Huangxu Li, Zheng Li, Zhenyu Zhang, Jiexin Zhu, Fei Guo, Xuan Gao, Zijuan Du, Jintao Chen, Tianlei Wang, Guanjie He, Ivan P. Parkin

**Affiliations:** ^1^ Christopher Ingold Laboratory Department of Chemistry University College London London WC1H 0AJ UK; ^2^ Electrochemical Innovation Lab (EIL) Department of Chemical Engineering University College London London WC1E 7JE UK; ^3^ Jiangsu Key Laboratory of Electrochemical Energy Storage Technologies College of Materials Science and Technology Nanjing University of Aeronautics and Astronautics Nanjing 210016 P. R. China; ^4^ Department of Chemistry City University of Hong Kong Kowloon, Hong Kong 999077 P. R. China; ^5^ School of Metallurgy and Environment Central South University Changsha 410083 P. R. China

**Keywords:** Additive, Aqueous Electrolyte, Dendrite Growth, Zinc Anode, pH Buffer

## Abstract

Aqueous zinc‐ion batteries have drawn increasing attention due to the intrinsic safety, cost‐effectiveness and high energy density. However, parasitic reactions and non‐uniform dendrite growth on the Zn anode side impede their application. Herein, a multifunctional additive, ammonium dihydrogen phosphate (NHP), is introduced to regulate uniform zinc deposition and to suppress side reactions. The results show that the NH_4_^+^ tends to be preferably absorbed on the Zn surface to form a “shielding effect” and blocks the direct contact of water with Zn. Moreover, NH_4_^+^ and (H_2_PO_4_)^−^ jointly maintain pH values of the electrode‐electrolyte interface. Consequently, the NHP additive enables highly reversible Zn plating/stripping behaviors in Zn//Zn and Zn//Cu cells. Furthermore, the electrochemical performances of Zn//MnO_2_ full cells and Zn//active carbon (AC) capacitors are improved. This work provides an efficient and general strategy for modifying Zn plating/stripping behaviors and suppressing side reactions in mild aqueous electrolyte.

## Graphical abstract

A multifunctional additive, NH_4_H_2_PO_4_, is proposed to regulate uniform zinc deposition and to suppress side reactions in a dilute aqueous electrolyte. Due to the pH buffer chemistry of NH_4_^+^ and (H_2_PO_4_)^−^ and the “shielding effect” of NH_4_^+^, promoted Coulombic efficiencies and lifespans of Zn based symmetric/asymmetric cells can be achieved.

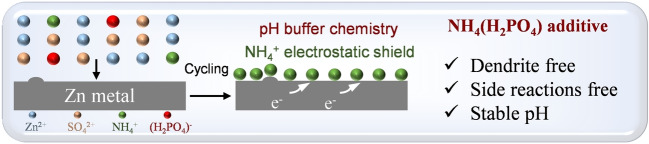

## Introduction

Contemporarily, the development of reliable energy storage systems (ESSs) has become a crucial task to build a greener world.^[1]^ The currently prevailed lithium‐ion batteries (LIBs) stand out as the dominant ESSs while still face tremendous challenges such as inflammability, high cost, and underlying political disputes.^[2]^ Although sodium‐ion batteries are emerging as an alternative to LIBs regarding cost‐effectiveness,^[3]^ it is difficult to address the intrinsic safety issue of organic electrolytes. Compared with them, aqueous zinc batteries (AZBs) attract great attention in ESSs on account of high safety, fast ionic conductivity of aqueous electrolyte, lost cost, high theoretical capacity (gravimetric: 820 mAh g^−1^; volumetric: 5851 mAh mL^−1^) and good compatibility of zinc anode with the aqueous electrolyte.^[4]^ Recently, enormous efforts have been dedicated on cathode materials mainly including Prussian blue analogues,^[5]^ V‐based oxides,^[6]^ and Mn‐based oxides.^[7]^ Nevertheless, the poor reversibility and inferior cycling stability of Zn anode hamper the future application of AZBs. To illustrate, analogous to other metallic anodes (e.g., Li, Na, and K, *etc*.), Zn anode suffers from severe dendrite growth stemming from inhomogeneous electric field distribution and the notorious short circuit issue is thus made; it is also faced with the inevitable hydrogen evolution reaction (HER) due to the more negative redox potential of Zn^2+^/Zn than that of HER, which induces the formation of porous zinc hydroxide and/or Zn_4_SO_4_(OH)_6_ ⋅ *x*H_2_O and aggravates the chemical corrosion.[^4b^, ^8^] These aspects are regarded as the main reasons for the poor Coulombic efficiency and short lifespan, which should be addressed simultaneously but are full of great challenges.^[9]^

A plethora of intriguing strategies have been proposed to achieve this target: i) coating artificial interface layers^[10]^ to avoid the direct contact between the electrolyte and zinc electrode; ii) guiding preferred crystal plane growth with a favorable lattice match;^[11]^ iii) constructing three‐dimensional host;^[12]^ iv) alloying with other metals;^[13]^ v) electrolyte engineering,^[14]^ *etc*. Among them, “water in salt” electrolyte design seems to be an efficient way to reduce water contents in Zn^2+^ solvation sheath but huge challenges come with consideration of the increased cost of high salt concentration as well as high viscosity.[^14f^, ^15^] In contrast, mixing some additives into dilute aqueous electrolyte (e.g., 1 M ZnSO_4_) is considered as a more promising strategy with regard to the facile process, cost‐effectiveness, and broad availability without compromising energy density.[^14d^, ^16^] However, most additives can only display one function for zinc anode protection. For instance, Zhu et al.^[17]^ introduced a TBA_2_SO_4_ additive into 2 M ZnSO_4_ electrolyte; they proved that TBA^+^ ions were absorbed on the zinc metal surface to form a shielding effect, which suppressed the dendrite growth. Very recently, Huang et al.^[14e]^ reported a La(NO_3_)_3_ additive for aqueous ZnSO_4_ electrolyte. It turned out that La^3+^ could reduce the repulsive force of the electric double layer to lead to the favorable electrodeposition of zinc metal. Chao et al.^[14c]^ utilized a glucose additive to replace one water molecule from the solvation structure of Zn^2+^ thus the decreased water activity could retard the side reaction. Anyway, the monofunctional additives can only provide a limited protection for zinc anode from a single aspect and it is of enormous significance to develop multifunctional additives to comprehensively protect the zinc anode.

**Figure 1:**
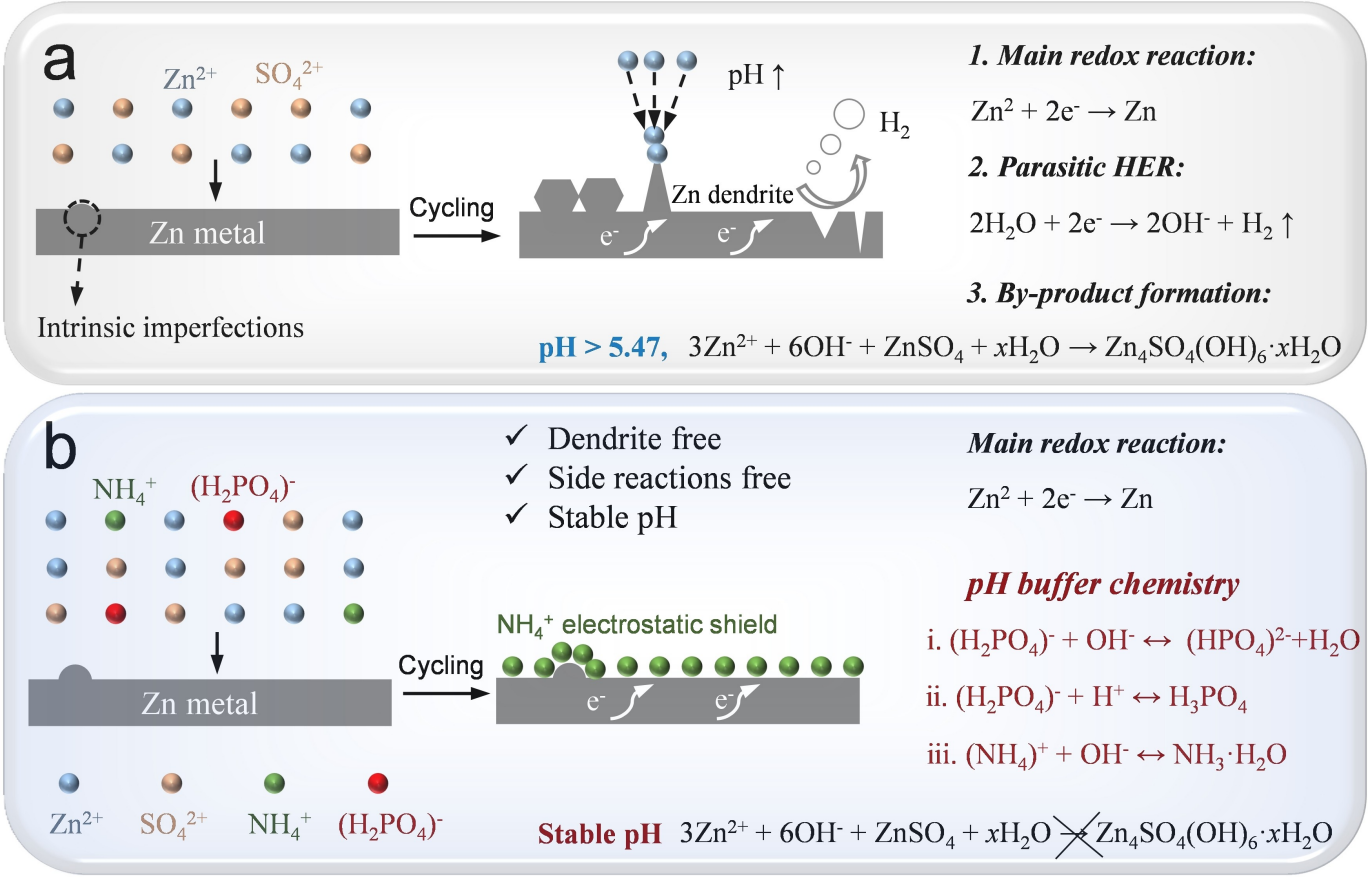
The schematic diagram of zinc plating processes in a) 1 M ZnSO_4_ (BE) and b) 1 M ZnSO_4_+25 mM NHP (DE).

Moreover, the interfacial proton concentration (pH) plays a pivotal role in zinc electrodeposition.^[18]^ Under the alkaline environment, zinc electrode would be passivated by the insulating ZnO and Zn(OH)_2_ byproducts along with severe Zn dendrite growth, which account for its irreversibility.[^4b^, ^19^] In contrast, within the neutral or mild acidic aqueous electrolyte (e.g., 1 M ZnSO_4_), Zn anode performs much enhanced reversibility with suppressed byproduct formation and less Zn dendrite growth. The pH value of the reported mild aqueous electrolyte of AZBs is normally ≈4.^[4b]^ As we mentioned above, HER is an inevitable reaction due to the intrinsic properties of water and zinc anode upon AZBs cycling, which usually increases the local pH values (higher OH^−^ concentration) and thus aggravates the formation of the porous and inert Zn_4_SO_4_(OH)_6_ ⋅ *x*H_2_O byproduct (when pH >5.47; Figure 1a).^[20]^ Accordingly, this high surface area byproduct incurs a non‐homogeneous zinc surface, which continuously consumes a great deal of Zn^2+^ and aggravates dendrite formation. As a consequence, a low CE and a short battery lifespan are obtained. Therefore, it is rather intriguing but is rarely investigated to stabilize the interfacial pH around Zn within an appropriate range for advantageous zinc deposition. The current electrolyte additive works have made great progress but mainly focused on changing Zn solvation sheath, forming SEI layers, lowering desolvation energy and guiding deposited preferable Zn lattice planes.[^16b^, ^21^] pH changes in the electrolyte and methods to control stable electrode‐electrolyte interphases in AZBs still need to be well conducted by properly selecting and understanding pH buffer additives.

Inspired by these two aspects, herein, we propose a multifunctional additive, ammonium dihydrogen phosphate (NHP), into a dilute aqueous ZnSO_4_ electrolyte to buffer the electrolyte pH value, to regulate highly reversible Zn plating/stripping and to suppress side reactions upon battery cycling. Both experimental and theoretical approaches disclosed that the zincophilic NH_4_^+^ tends to be absorbed on Zn electrode surface and thus the “tip effect” was shielded and water molecules were excluded from the direct contact with Zn, which achieved dendrite‐free zinc deposition and a suppressed hydrogen evolution reaction. More significantly, the pH buffer NH_4_^+^ and (H_2_PO_4_)^−^ can maintain the concentrations of H^+^ and OH^−^ at the interface of Zn electrode and electrolyte, which realized a secondary protection for Zn electrodes (Figure 1b).[^20^, ^22^] As a consequence, the NHP additive that serves as a “shield” and a pH buffer enables highly reversible Zn plating/stripping behaviors: the Zn//Zn symmetric cell using NHP stably cycled 2100 h at 1 mA cm^−2^, 1900 h at 4 mA cm^−2^, and 930 h at 10 mA cm^−2^; the Zn//Cu asymmetric cell using NHP displayed a high average Coulombic efficiency of 99.4 % throughout 1000 cycles. Besides, the NHP additive improved the electrochemical performances of Zn//MnO_2_ full cells and Zn//active carbon (AC) capacitors. This work provides a general strategy for modifying Zn plating/stripping behaviors and excluding side reactions in mild aqueous electrolyte from a pH buffer perspective.

## Results and Discussion

### pH buffer chemistry

**Figure 2:**
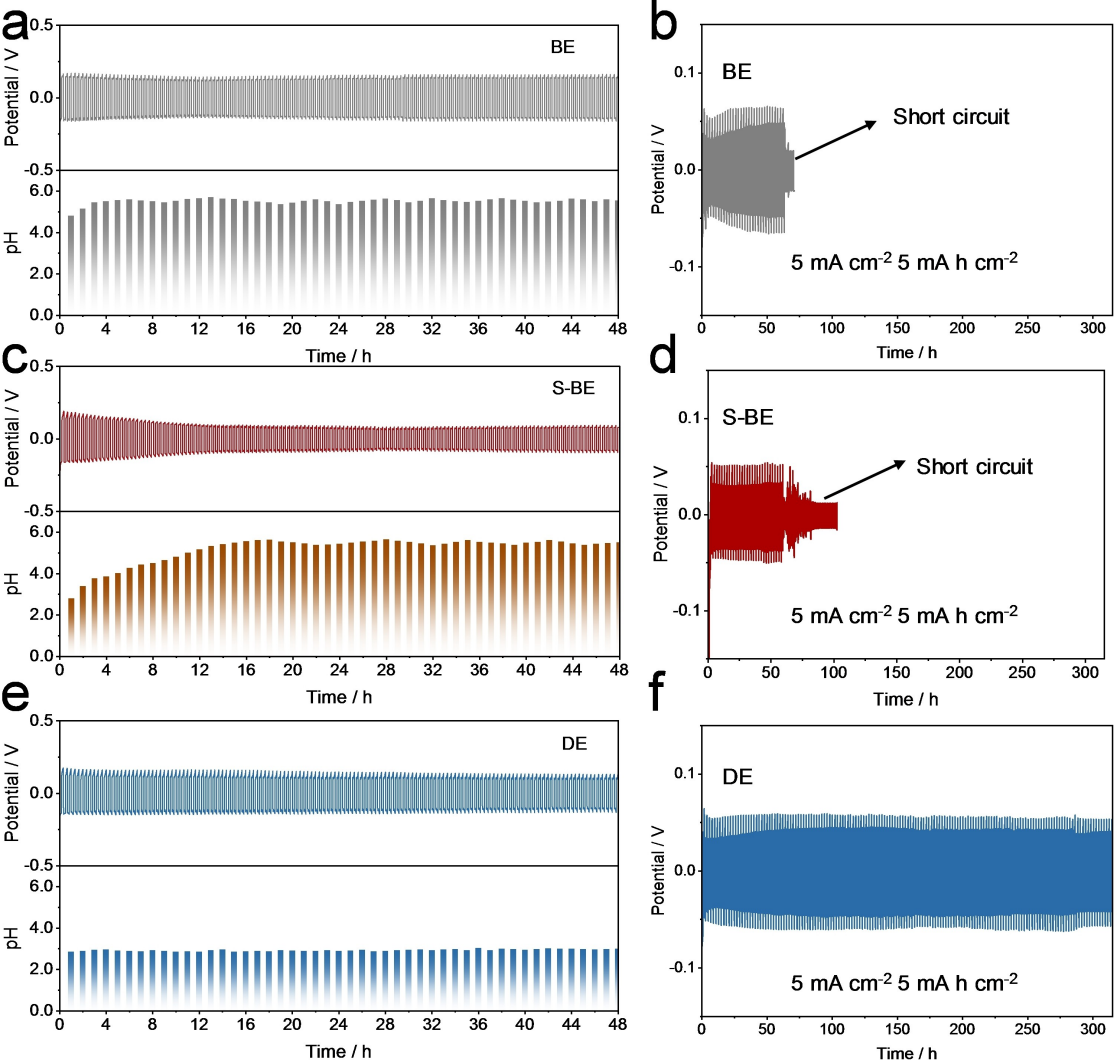
pH buffer chemistry. In situ pH monitoring of a) 1 M ZnSO_4_ (BE), c) 1 M ZnSO_4_+*x*H_2_SO_4_ (S‐BE), and e) 1 M ZnSO_4_+25 mM NHP (DE) within Zn//Zn symmetric cells upon cycling at 10 mA cm^−2^. Cycling performances of Zn//Zn symmetric cells in b) 1 M ZnSO_4_ (BE), d) 1 M ZnSO_4_+*x*H_2_SO_4_ (S‐BE), and f) 1 M ZnSO_4_+25 mM NHP (DE) at 5 mA cm^−2^/5 mAh cm^−2^.

To begin with, the pH values of 1 M ZnSO_4_ (BE) and 1 M ZnSO_4_+25 mM NHP (DE) were in situ monitored upon the Zn//Zn symmetric cell cycling at 10 mA cm^−2^. As mentioned earlier, HER will inevitably occur when H_2_O/H^+^ directly contacts with Zn surface during AZBs cycling, which results in the Zn electrode corrosion and a rise of local OH^−^ concentration as shown in Figure 1a. The increased OH^−^ reacts with Zn^2+^ and SO_4_^2−^ to form the porous and inactive Zn_4_(OH)_6_(SO_4_) ⋅ 5H_2_O on the Zn surface, which in turn deteriorates dendrite growth and blocks the charge transfer process.^[23]^ As observed, the pH values of BE remarkably increased from the initial 4.29 to 5.6 only within 6 h (Figure 2a) and fluctuated in the subsequent cycles. The increased pH values during first cycles can be ascribed to HER. While the fluctuated pH values in the succeeding cycles could be due to the competing reaction between HER (increasing pH) and Zn_4_(OH)_6_(SO_4_) ⋅ 5H_2_O formation (decreasing pH).[^2a^, ^4b^] In sharp contrast, the pH values of DE remained pretty steady under the same condition (Figure 2e). This result reveals that NHP serves as a pH buffer by virtue of NH_4_^+^ and (H_2_PO_4_)^−^, which is capable of stabilizing electrode‐electrolyte interphases and suppressing the side reactions (Figure 1b).^[24]^ Besides, on the purpose of avoiding the influence of the initial pH value, the pH value of another BE was regulated to be ≈2.8 (similar to that of DE) by a small amount of H_2_SO_4_ (denoted as S‐BE). In Figure 2c, the pH values of S‐BE increased from 2.8 to 5.63 within 18 h at 10 mA cm^−2^ and remained fluctuated in the subsequent cycles, which can also be attributed by the competing reaction between Zn_4_(OH)_6_(SO_4_) ⋅ 5H_2_O formation (decreasing pH) and HER (increasing pH). To further confirm the effectiveness of this pH buffer, Zn//Zn symmetric cells in these three electrolytes were tested at 5 mA cm^−2^/5 mAh cm^−2^. In Figure 2b and 2d, Zn//Zn symmetric cells using BE and S‐BE displayed a similar short lifetime of ≈60 h. Remarkably, the cell could sustain a much longer cycle time of 315 h with the assistance of NHP (Figure 2f) under such a harsh condition. As illustrated in Figure 1b, when the local OH^−^ concentration increases, the (H_2_PO_4_)^−^ and (NH_4_)^+^ can react with the increased OH^−^; while the local H^+^ concentration increases, the (H_2_PO_4_)^−^ is capable of reacting with the increased H^+^. With stable pH values, the Zn_4_SO_4_(OH)_6_ ⋅ 5H_2_O byproduct will not be formed and thus good electrochemical performances could be achieved. The above results verified that the NHP additive can stabilize the pH of the baseline electrolyte upon cycling, which is not to do with the initial pH value.

### Zn Plating/Stripping Morphologies

**Figure 3:**
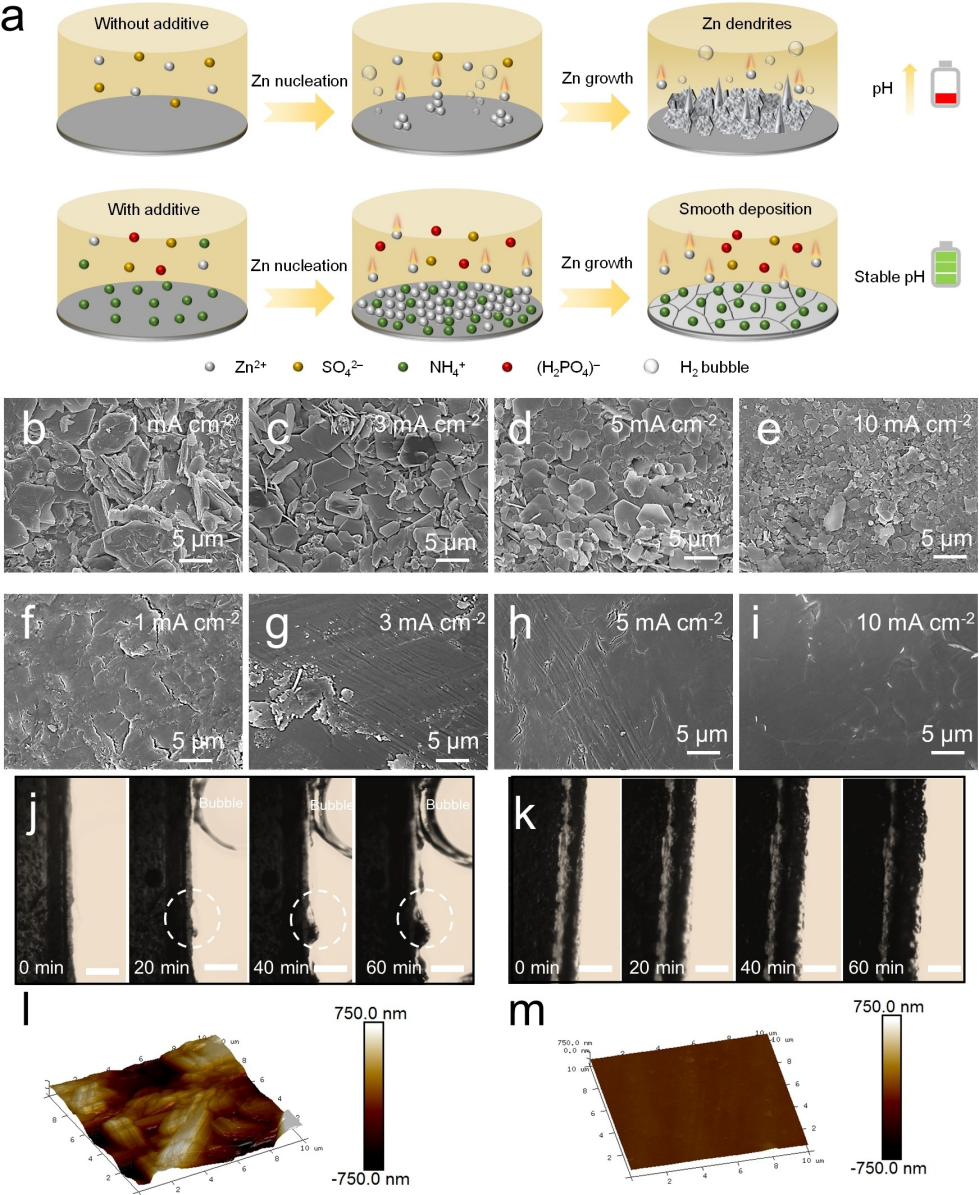
Zn plating/stripping morphologies. a) Schematic illustration of the effect of NHP additives on the Zn deposition process. SEM images of Zn deposits on a Zn substrate (Zn//Zn symmetric cells) at various current densities from 1 mA cm^−2^ to 10 mA cm^−2^ with 1 mAh cm^−2^ in b–e) 1 M ZnSO_4_ (BE) and f–i) 1 M ZnSO_4_+25 mM NHP (DE). In situ optical microscopy images of Zn plating behaviors on a Zn substrate at 10 mA cm^−2^ in j) 1 M ZnSO_4_ (BE) and k) 1 M ZnSO_4_+25 mM NHP (DE), scale bar: 300 μm. AFM images of cycled Zn electrodes in l) 1 M ZnSO_4_ (BE) and m) 1 M ZnSO_4_+25 mM NHP (DE).

Scanning electron microscopy (SEM) was then carried out to probe how the NH_4_H_2_PO_4_ (NHP) additive affects the Zn electrodeposition behaviors at various current densities from 1 mA cm^−2^ to 10 mA cm^−2^ with a constant capacity of 1 mAh cm^−2^ in DE and BE, respectively. As shown in Figure 3b–3e, all SEM images exhibited quasi‐hexagonal platelets of Zn deposits, which is consistent with previous aqueous ZnSO_4_ related works on account of a lower thermodynamic free energy of the exposed (002) plane.^[14e]^ Besides, in accordance with previous reports, the nucleation size of Zn is decreased while the nucleation gets increasingly compact with increasing the current density.[^2a^, ^14e^, ^25^] According to previous works,[^25b^, ^25c^] it has been verified that nuclei size is proportional to the inverse of overpotential while an increasing current density leads to a higher nucleation overpotential, which explains this phenomenon. Even under a high current density of 10 mA cm^−2^, the deposited platelets were dispersed. In sharp contrast, with the assistance of the NHP additive, the zinc deposits display smooth and dense morphologies at different current densities (Figure 3f–3i), which are analogous to the original zinc foil surface (Figure S1). The same are true with the cycled Zn anodes after longer cycles (Figure S2). Energy dispersive spectrometry (EDS) was further conducted to characterize the elemental composition of the cycled zinc electrode. As presented in Figure S3, S and O elements can be clearly found within the sample under BE, demonstrating that some SO_4_^2−^ contained byproducts were formed upon battery cycling. On the contrary, there is no S element signal in the sample under DE (Figure S4), indicative of good inhibitory effect of the NHP additive on some common side reactions of AZBs.

Moreover, in situ optical microscopy was performed to visually observe the Zn electrodeposition behaviors and corresponding interfacial reactions with electrolytes at 10 mA cm^−2^. With time, as shown in Figure 3j, a H_2_ bubble and zinc dendrites gradually evolved into larger sizes at the zinc anode surface within 60 min in BE. However, the DE sample exhibited a totally opposite result: nearly no bubbles and zinc dendrites could be found in Figure 3k with much flatter zinc deposits under the same condition. The same are true with the results of the atomic force microscopy (AFM) in Figure 3l and 3m: the presence of the NHP additive significantly reduced the roughness of the cycled zinc anode surface. The above analyses accordingly confirmed that NHP is capable of reducing side reactions and mitigating zinc dendrite growth as illustrated in Figure 3a, which enhances the reversibility of zinc anode.

### Corrosion Suppression

Different concentrations of NHP (0, 10 mM, 25 mM, and 50 mM) were added into the baseline 1 M ZnSO_4_ electrolyte. All of them appear homogeneous phase as seen in Figure S5. In Figure S6, it can be clearly found that the pH values of the electrolytes become lower with increasing the concentrations of NHP additives, which will be discussed later on. FT‐IR and Raman spectra were collected to confirm if the NHP additives could affect the solvation structure of Zn^2+^. It turned out that there are no obvious differences among Figure S7 and Figure S8 for all NHP containing electrolytes and BE, unlike previous additive works, demonstrating NHP additive has no impact on the Zn^2+^ solvation structure.[^14c^, ^26^] In addition, Zn//Cu asymmetric cells were assembled to determine the optimal concentration of the NHP additive. As presented in Figure S9, the Zn//Cu asymmetric cell with 25 mM NHP stands out as the best one with a highest average Coulombic efficiency (CE) of 99.4 % and a longest lifespan of 1000 cycles, which outweigh those of many previous works.[^14c^, ^23^, ^24^, ^27^] A higher CE is a powerful indicator of better striping/plating behaviors of metal electrodes.^[14d]^ In contrast, the Zn//Cu asymmetric cells with 10 mM NHP and 50 mM NHP displayed average Coulombic efficiencies of 98.9 % (450 cycles) and 99 % (500 cycles), respectively. The above analyses confirm that 1 M ZnSO_4_+ 25 mM NHP electrolyte (hereafter DE) is the optimal choice of this work.

**Figure 4:**
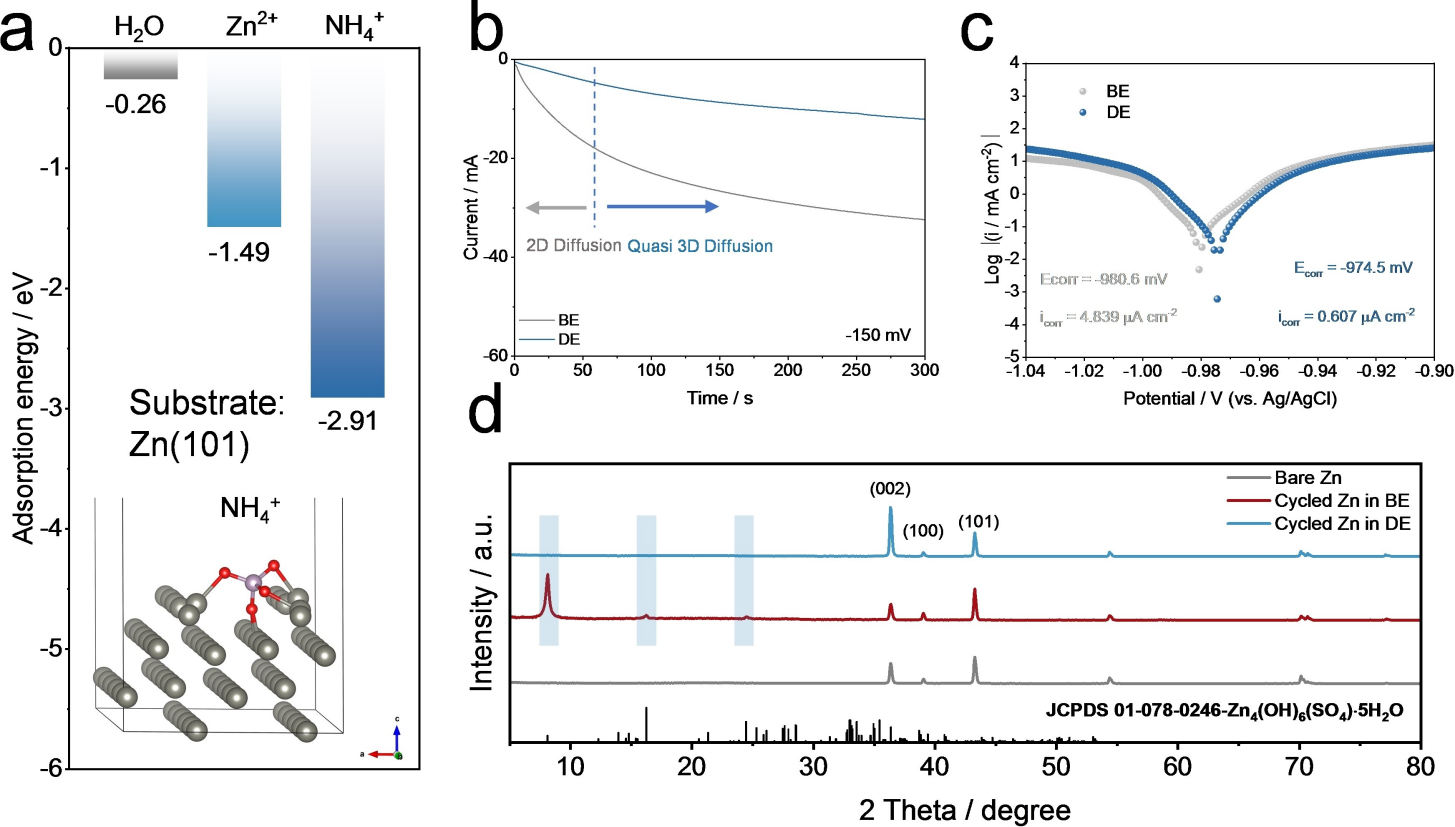
Corrosion suppression. a) The adsorption energy between H_2_O/Zn^2+^/NH_4_^+^ on the Zn (101) substrate (inset: the illustration of the NH_4_^+^ case). b) Chronoamperometry (CA) curves of Zn anodes at −150 mV. c) Linear polarization curves of Zn anodes. d) XRD patterns of pure Zn and cycled Zn anodes in 1 M ZnSO_4_ (BE) and 1 M ZnSO_4_+25 mM NHP (DE), respectively.

A series of electrochemical measurements were conducted to further clarify the positive effects of the NHP additive on Zn electrode side reactions. Chronoamperometry (CA) is a widely used electrochemical approach to characterize the Zn nucleation and growth. It has been well established that a higher current response indicates a larger effective surface area.[^14e^, ^26^] In Figure 4b, when a normal overpotential (−150 mV) was applied, a rapid current response could be seen at the initial stage for both samples, which may be attributed to the Zn nucleation process. With the plating process continuing, the current response of the BE cell still increases, demonstrating the increased effective electrode area and thus accounting for the Zn dendrite formation. In sharp contrast, there was a relatively lower increase in current response for the DE cell. This result verified that a denser and smoother electrodeposition of Zn can be achieved with the assistance of NHP (Figure 3). Additionally, the linear polarization curves for both samples were presented in Figure 4c. Clearly, the corrosion current density of the Zn electrode in the DE decreases from 4.839 μA cm^−2^ to 0.607 μA cm^−2^ with the presence of NHP, which demonstrates that the corrosion was suppressed.[^14e^, ^28^]

In order to further decipher the underlying mechanisms, density functional theory (DFT) was performed to calculate the adsorption energies of H_2_O, Zn^2+^, NH_4_^+^ on the Zn (101) substrate. It turns out in Figure 4a that the adsorption energy of NH_4_^+^ (−2.91 eV) is much lower than those of H_2_O (−0.26 eV) and Zn^2+^ (−1.49 eV), indicating that NH_4_^+^ is preferred to be absorbed on the surface of Zn electrode to form a shield that blocks the direct contact of H_2_O and Zn. This explains the restrained corrosion and corresponds to the experimental results in Figure S10 even when a rest period was applied for Zn//Cu asymmetric cells. Figure S11 also confirmed an expanded electrochemical window of the electrolyte with assistance of NHP additives. In addition, 12.5 mM (NH_4_)_2_SO_4_ and 12.5 mM Zn(H_2_PO_4_)_2_ were separately added into the baseline 1 M ZnSO_4_ solution to probe the respective effects of NH_4_^+^ and (H_2_PO_4_)^−^ on the pH value. In Figure S13, the NH_4_^+^ drove the pH value from 4.27 (BE) down to 4.18, which may be due to the hydrolysis reaction of NH_4_^+^ in aqueous solution. With (H_2_PO_4_)^−^ additive, in Figure S14, the pH value of the electrolyte significantly decreased to 3.04, which corresponds to the proton dissociation of (H_2_PO_4_)^−^ into (HPO_4_)^2−^. Additionally, in Figure S15–S16, the Zn//Cu cells and the Zn symmetric cells that contain NH_4_^+^ or (H_2_PO_4_)^−^ exhibited improved CEs and cycle lifespans, indicative of the positive effects of NH_4_^+^ and (H_2_PO_4_)^−^ on the uniform Zn stripping/plating.

In Figure 4d, the XRD patterns were collected on the bare Zn foil, cycled Zn in BE and cycled Zn in DE, which showed that after cycling in BE, intense impurity peaks could be found, indicating that a plethora of Zn_4_(OH)_6_(SO_4_) ⋅ 5H_2_O (ZHS) was generated on the Zn surface, which is in agreement with Figure 1 and 3. On the contrary, all of the diffraction peaks of the cycled Zn in DE were indexed to pure Zn and no ZHS signal was observed, which further confirms the positive effect of NHP on the pH regulation. It should be noted that the intensity of Zn (002) crystal plane in the DE case became dominant, which has been proved to be favorable for the Zn electrodeposition and the suppressed dendrite growth.^[11b]^ But the surface‐preferred Zn (101) in bare Zn and in the cycled Zn in BE was thought to deteriorate Zn nucleation and facilitate dendrite growth, which is in line with Figure 3.[^11b^, ^11c^] From the above analyses, one can believe that NHP additive as the pH buffer and the shield can suppress the side reactions and ameliorate Zn electrodeposition behaviors.

### Zn Plating/Stripping Behaviors

**Figure 5:**
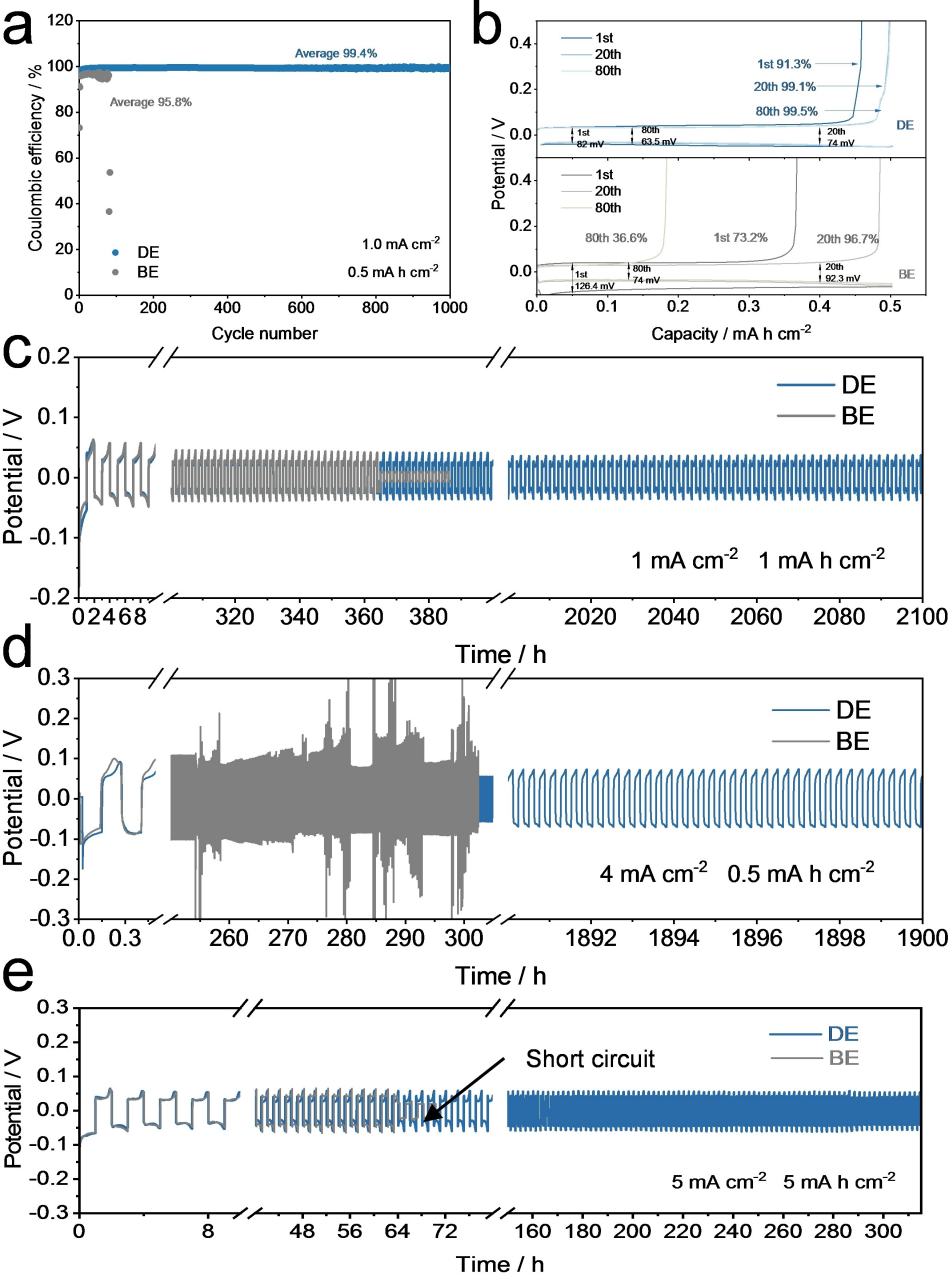
Zn plating/stripping behaviors under different electrolytes. a) Coulombic efficiency of Zn//Cu asymmetric cells during long‐term cycling and b) corresponding voltage profiles at different cycles. Plating/stripping cyclabilities of Zn symmetric cells at c) 1 mA cm^−2^ with a capacity of 1 mAh cm^−2^, d) 4 mA cm^−2^ with a capacity of 0.5 mAh cm^−2^, and e) 5 mA cm^−2^ with a capacity of 5 mAh cm^−2^.

The outstanding capability of the NHP pH buffer additive on the Zn dendrite suppression has been verified via previous theoretical and experimental analyses. In this section, Zn plating/stripping behaviors on Zn/Cu substrates with the assistance of the NHP additive were further analyzed. To begin with, the reversibility of Zn plating/stripping behaviors was tested on the basis of Zn//Cu asymmetric cells at 1 mA cm^−2^ with 0.5 mAh cm^−2^ (cut‐off voltage: 0.5 V). As presented in Figure 5a and 5b, during the first cycles, Zn in BE displayed relatively low CEs, corresponding to the poor initial lattice fitting stage of Zn on Cu sheet in BE.^[14c]^ And the Zn//Cu cell in BE only showed a limited lifespan of ≈80 cycles with a low average CE of 95.8 % due to the aforementioned dendrite formation and side reactions. In sharp contrast, the Zn//Cu cell in DE displayed a much improved initial CE of 91.3 % and a high average CE of 99.4 % throughout 1000 cycles, which again proved that NHP additive is capable of suppressing Zn dendrite growth and avoiding those notorious parasitic reactions as discussed in Figure 3 and Figure 4. Furthermore, the Zn//Cu cells were subjected to a harsher condition of 2 mA cm^−2^ and 1 mAh cm^−2^ (Figure S17). Likewise, the Zn//Cu battery using DE presented a higher initial CE of 93.0 %, a longer lifetime and a significantly enhanced average CE of 99.5 % throughout 800 cycles; while the control group failed at 59^th^ cycle with a low average CE of 94.7 %. The same is also true with the Zn//Zn symmetric cell configuration under various test conditions. As displayed in Figure 5c, Figure S18, Figure S19, and Figure S20, the Zn//Zn symmetric cells using DE are able to stably cycle 2100 h, 1750 h, 1350 h, and 930 h at 1 mA cm^−2^, 2 mA cm^−2^, 5 mA cm^−2^ and even up to 10 mA cm^−2^ with a fixed areal capacity of 1 mAh cm^−2^, respectively, which are much better than the Zn//Zn symmetric cells using BE in the same conditions with lifespans of 365 h, 248 h, 110 h, and 67 h. The Zn//Zn symmetric cells using DE can also run 1900 h at 4 mA cm^−2^ and 0.5 mAh cm^−2^ while the BE case encountered a severe short circuit after ≈250 h (Figure 5d). Figure S21 verified a good rate property of the Zn plating/stripping in DE. More fascinatingly, under a relatively practical condition of 5 mA cm^−2^ and 5 mAh cm^−2^, in Figure 5e, the Zn//Zn symmetric cells using DE still revealed a highly reversible Zn plating/stripping behavior of 315 h, which surpass 64 h for the BE counterpart and even outweigh many previous reports.[^14c^, ^14e^, ^27^] Based on a powerful protocol by Zhi et al.,^[8]^ Figure S22 presented an excellent cycling stability of the Zn//Zn cell using DE with the combination of cycling properties and the electrochemical impedance spectra (EIS) analysis. These results strongly support the fact that the NHP additive leads to a uniform Zn electrodeposition and suppressed side reactions as observed in Figure 3 and Figure 4.

### Electrochemical Performances of Zn‐Based Full Cells

**Figure 6:**
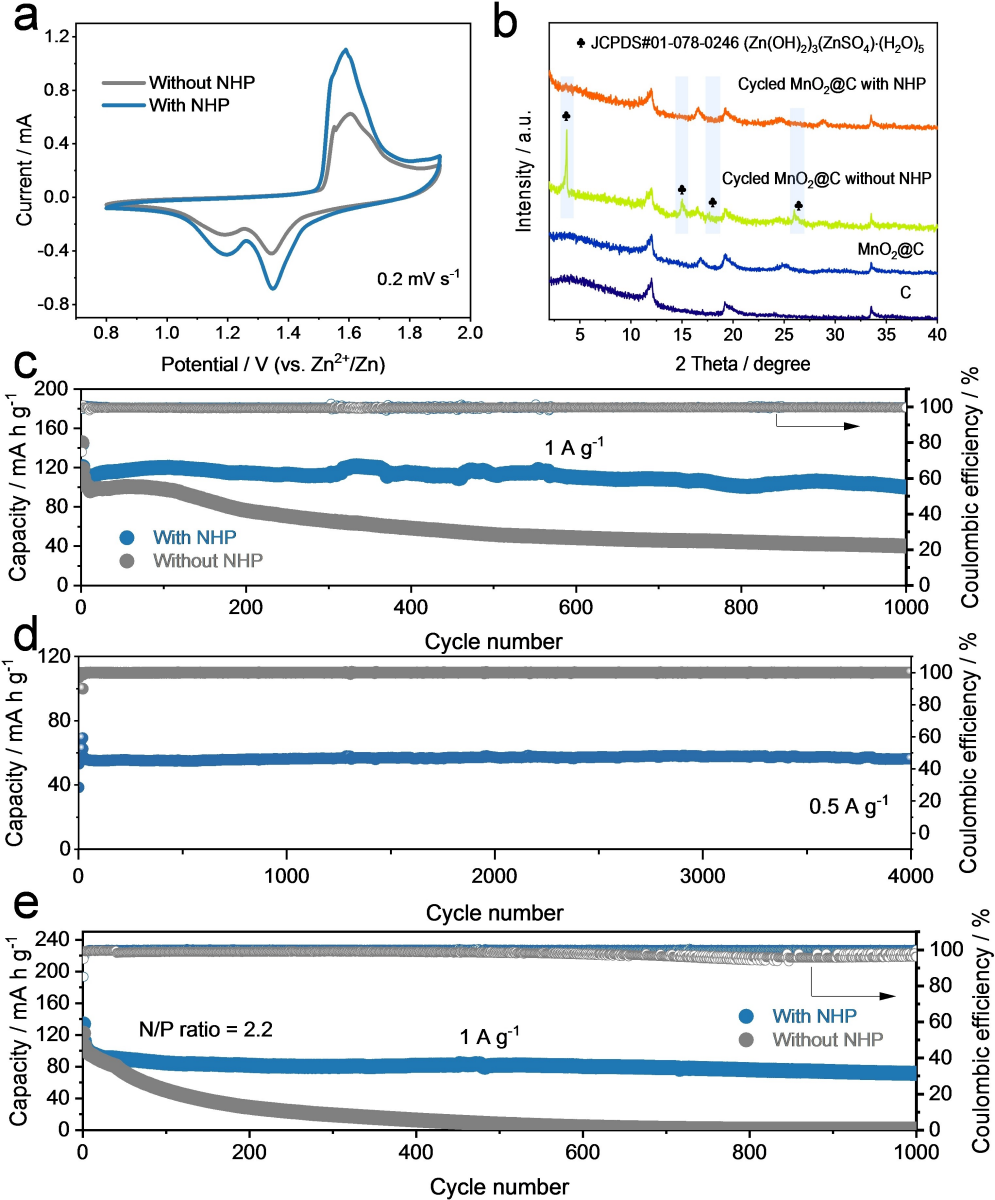
Electrochemical performances of full cells. a) CV curves. b) XRD patterns (Mo K_α_ radiation) of carbon paper, fresh MnO_2_@carbon paper cathode, cycled MnO_2_@carbon paper cathode with/without NHP. Long‐term cycling properties of c) Zn//MnO_2_ full cells at 1 A g^−1^ and d) the Zn//AC capacitor with NHP at 0.5 A g^−1^ in a large N/P ratio. e) Cycling performances of Zn//MnO_2_ cells at 1 A g^−1^ in a low N/P ratio of 2.2.

With more practical concerns, Zn metal anode was directly coupled with the commercial MnO_2_ (without further modification; the XRD pattern is presented in Figure S23) cathode to fabricate the Zn//MnO_2_ full cell with/without NHP. In general, the addition of a small amount of NHP additives enables effective performance improvement of the Zn//MnO_2_ full cell. In Figure 6a, the cyclic voltammetry (CV) curves and peak positions of both samples are almost identical, which indicates the NHP additive has negligible influence on the kinetics of the Zn//MnO_2_ full cell. This can be supported by electrochemical impedance spectroscopy (EIS) results of Figure S24 and the rate performances in Figure S25. More importantly for the cycling property, as displayed in Figure 6c, the Zn//MnO_2_ full cell with NHP is capable of maintaining a much better cycling stability (higher capacity retention) for 1000 cycles than the control group counterpart at 1 A g^−1^, which is in agreement with the good pH buffer capability of NHP additive as discussed in Figure 1 and 2. According to the previous reports, a stable pH value could prevent the MnO_2_ cathode materials from the inactive ZHS layer.[^20^, ^22^, ^24^] Therefore, the XRD patterns of MnO_2_@carbon paper cathode after cycling with/without NHP were collected. Similar to the results in Figure 4d, obvious ZHS layer signals were found in cycled MnO_2_@C without NHP (Figure 6b) while almost no such peaks existed in cycled MnO_2_@C with NHP. These results again confirm the merits of NHP additives. Our strategy was also applied in the Zn//AC capacitors with the commercial activated carbon as the cathode in DE (Figure 6d). The capacitor was able to stably run 4000 cycles at a current density of 0.5 A g^−1^ with an ultrahigh average CE of 99.9 %, which demonstrates that the NHP additive can be applied to wide application areas regarding Zn metal anodes.^[29]^ Moreover, a low capacity ratio of negative electrode to positive electrode (known as N/P ratio) is of great importance for practical applications. In this regard, we fabricated the Zn//MnO_2_ and Zn//AC cells with lower N/P ratios to investigate the effectiveness of our strategy under harsher circumstances by electrochemically depositing suitable amounts of Zn on Cu substrates (i.e., anode‐less configuration).^[30]^ As shown in Figure 6e, the NHP additive enables the Zn//MnO_2_ cell (N/P ratio: 2.2) to significantly prolong the lifespan from 400 cycles to more than 1000 cycles at 1 A g^−1^ with much improved CEs and cycling stability. The Zn//AC capacitor with a low N/P ratio of 4 is also capable of stably cycling over 900 cycles (Figure S27). The above results again confirm the great promise of NHP additives.

## Conclusion

In summary, a cheap and efficient additive, ammonium dihydrogen phosphate, was introduced into a dilute aqueous ZnSO_4_ electrolyte to buffer the electrolyte pH value, to suppress dendrite growth and side reactions during cycling. Both experimental and theoretical results revealed that the zincophilic NH_4_^+^ was preferably absorbed on the Zn surface to construct a “shielding effect” and to block the direct contact of water with Zn, which achieved dendrite‐free zinc deposition and a suppressed hydrogen evolution reaction. More encouragingly, the pH buffer NH_4_^+^ and (H_2_PO_4_)^−^ maintained the concentrations of H^+^ and OH^−^ at the electrolyte‐electrode interface to build a secondary protection for Zn electrodes. Accordingly, the NHP additive enables highly reversible Zn plating/stripping behaviors: the Zn//Zn symmetric cell using NHP stably cycled 2100 h at 1 mA cm^−2^, 1900 h at 4 mA cm^−2^, and 930 h at 10 mA cm^−2^; the Zn//Cu asymmetric cell using NHP displayed a high average CE of 99.4 % within 1000 cycles. Furthermore, the electrochemical performances of Zn//MnO_2_ full cells and Zn//active carbon (AC) capacitors were boosted with assistance of NHP additive. Our work provides a general strategy for dendrite‐free Zn deposition and avoiding side reactions in mild aqueous zinc‐ion batteries.

## Conflict of interest

The authors declare no conflict of interest.

1

## Supporting information

As a service to our authors and readers, this journal provides supporting information supplied by the authors. Such materials are peer reviewed and may be re‐organized for online delivery, but are not copy‐edited or typeset. Technical support issues arising from supporting information (other than missing files) should be addressed to the authors.

Supporting Information

## Data Availability

The data that support the findings of this study are available from the corresponding author upon reasonable request.
