# Supplementary material for: Highly Reversible Zinc Metal Anode in a Dilute Aqueous Electrolyte Enabled by a pH Buffer Additive
Source: Angew Chem Int Ed Engl. 2022 Dec 7;62(5):e202212695. doi: 10.1002/anie.202212695 (PMC10107295; doi:10.1002/anie.202212695)
Supplement: Supplementary file 1 — Supporting Information [file ANIE-62-0-s001.pdf]

Angewandte Chemie
Eine Zeitschrift der Gesellschaft Deutscher Chemiker

Supporting Information

Highly Reversible Zinc Metal Anode in a Dilute Aqueous Electrolyte Enabled by a pH Buffer Additive

W. Zhang, Y. Dai, R. Chen, Z. Xu, J. Li, W. Zong, H. Li, Z. Li, Z. Zhang, J. Zhu, F. Guo, X. Gao, Z. Du, J. Chen, T. Wang, G. He, I. P. Parkin**

1. *Electrolyte and electrode preparation*

The baseline 1 M $ZnSO_4$ electrolyte (BE) was prepared by dissolving 0.25 mol $ZnSO_4 \cdot 7H_2O$ (>99%, VWR chemicals) into 250 mL deionized water with constant magnetic stirring for 30 min. The designed electrolytes were then prepared by mixing proper amounts of $NH_4H_2PO_4$ (NHP; ≥ 99.5%, Sigma-Aldrich) with suitable amounts of the as-prepared 1 M $ZnSO_4$ solution to control the concentrations of NHP additives as 10 mM, 25 mM, and 50 mM. The optimized concentration of NHP is 25 mM and its corresponding electrolyte is denoted as DE. The MnO_2 cathodes consisted of 70 wt.% commercial MnO_2 powder (precipitated active for synthesis, Sigma-Aldrich), 20 wt.% acetylene black (battery grade, MTI), and 10 wt.% poly(vinylidene fluoride) (PVDF; average M_w ~534000, Sigma-Aldrich) with N-methyl-2-pyrrolidine (NMP; ≥ 99%, Sigma-Aldrich) as the solvent. Carbon paper (hydrophilic type; TORAY) was selected as the current collector. Active carbon (YP-80F, Kuraray Chemical) electrodes were prepared by the same method.

2. *Materials Characterizations*

SEM was conducted on JEOL-JSM-6700F (Voltage: 5 kV, emission current: 110.8 μA) under a low vacuum of < 3×10^{-4} Pa. The *in-situ* optical microscope was conducted on VisiScope® BL254 T1 (VWR) instrument with a specially designed electrolytic cell in a Zn//Zn symmetric configuration. A Bruker dimension Icon with Scanasyst device was employed to conduct AFM experiments. The XRD patterns of Zn foils were performed on a PANalytical Empyrean device (Cu K_α radiation; 40 kV, 40 mA) within 5~80 ° with

a step size of 0.05 ° and a scan rate of 0.1 ° s^{-1}. The XRD pattern of MnO_2 was collected on a STOE SEIFERT diffractometer with Mo K_{α} radiation (50 kV, 30 mA) within 2~40 ° to decrease the impact of manganese atoms fluorescent effect in the X-rays produced by a copper target.[1] A LabRAM HR Evolution instrument (laser wavelength: 532 nm) was carried out to collect the Raman spectra. AZ9861 pH meter (AZ Instrument Corp.) was performed to monitor the pH values. Contact angles were acquired on Kruss DSA25E (Germany).

3. *Electrochemical Measurements*

The Zn//Zn symmetric cells, Zn//Cu cells, Zn//MnO_2 full cells and Zn//AC capacitor were assembled to evaluate the electrochemical performances based on CR2032 coin cell with glass fiber (Whatman GF/A) as the separator and with/without additives in Neware battery test system (Shenzhen, China). For Zn//MnO_2 full cells, 0.1 M $MnSO_4$ was added into the electrolyte to suppress Mn dissolution (1 M $ZnSO_4$+0.1 M $MnSO_4$ and 1 M $ZnSO_4$+0.1 M $MnSO_4$+25 mM NHP). The Zn//MnO_2 full cells were pre-cycled for 10 cycles at 0.2 A g^{-1} before rate performance and long-term cycling. The mass loading of MnO_2 and AC is 1.5 mg cm^{-2}. The amounts of electrolytes for each cell were controlled as 100 μL. The thickness of Zn anode is 100 μm. To realize low N/P ratios in full cells, a suitable amounts of Zn on Cu substrate from the electrochemical deposition approach was used to match the cathode with a known areal capacity. The mass loading of thick MnO_2 and thick AC is ~8 mg cm^{-2} and ~5 g cm^{-2}, respectively. A Biologic VMP-3 electrochemical workstation was carried out to conduct EIS within the

frequency range of 10^5 ~ 10^{-2} Hz, to perform chronoamperogram (CA) of zinc symmetric cells at an overpotential of -150 mV, linear polarization curves at 1 mV s^{-1} with Zn plate as the working electrode and the counter electrode and Ag/AgCl as the reference electrode,[2] LSV curves based on Zn//Ti half cells at 10 mV s^{-1}.

4. *Computational methods and models*

All calculations were carried out by using the projector augmented wave method in the framework of the density functional theory (DFT),[3] as implemented in the QUANTUM ESPRESSO.[4] The generalized gradient approximation (GGA) and Perdew–Burke–Ernzerhof (PBE) exchange functional[3] was used. The plane-wave energy cutoff was set to 38 Ry, and the Monkhorst–Pack method[5] is employed for the Brillouin zone sampling. The convergence criteria of energy and force calculations were set to 10^{-5} Ry/atom and 0.01 Ry/Å, respectively. The Zn surface model was built by the 2×6 supercell of Zn (101). A vacuum region of 15 Å is applied to avoid interactions between the neighboring configurations. DFT-D2 method was used to account for the vdW interactions between Zn (101) surface and molecule (ion).[6] To explore the interactions between the molecule (ion) and Zn surface, the adsorption energies of the molecule (ion) adsorption on Zn (101) surface were calculated. Here, the adsorption energies (E_{ad}) were calculated by the energy difference of the system after and before adsorption:[7] E_{ad}= E(Ion adsorbed Zn) - E(Ion) - E(Zn), where E(Ion adsorbed Zn), E(Ion) and E(Zn), represent the DFT energies of the ion or molecule

adsorbed Zn surface, the energy of an ion or molecule, and the energy of clean Zn surface, respectively.

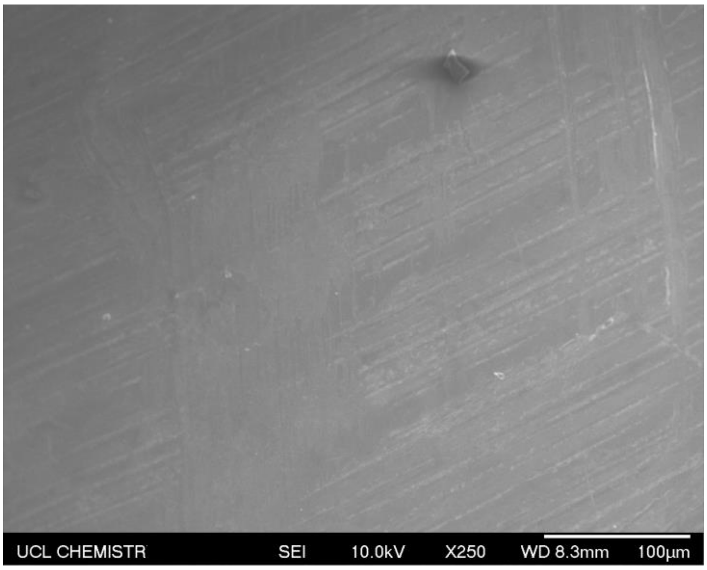

Figure S1. SEM image of the pristine zinc foil (top view).

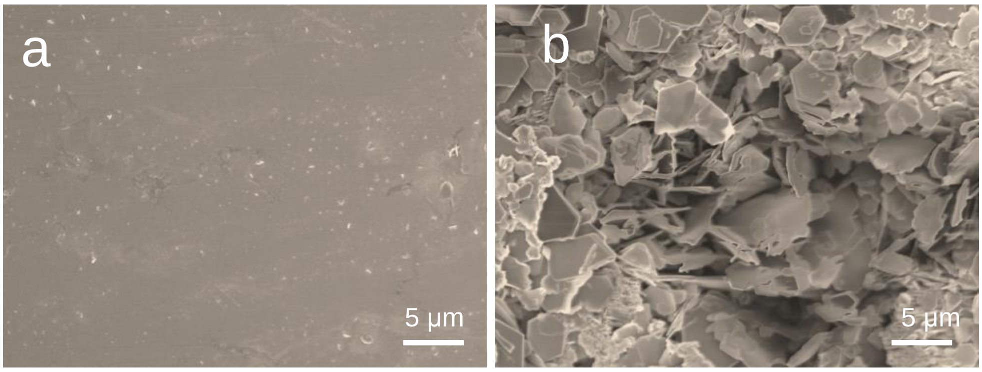

Figure S2. SEM images of Zn deposits on a substrate (Zn//Zn symmetric cells) at 5 mA cm^{-2} and 1 mA h cm^{-2} for 100 cycles in a) 1 M $ZnSO_4$ + 25 mM NHP (DE) and b) 1 M $ZnSO_4$ (BE).

Similar to Figure 3b-3e, Figure S2b shows quasi-hexagonal platelets of Zn deposits in BE and the deposited platelets were scattered. In contrast, the Zn deposits in DE still displayed a smooth and dense morphology even after 100 cycles.

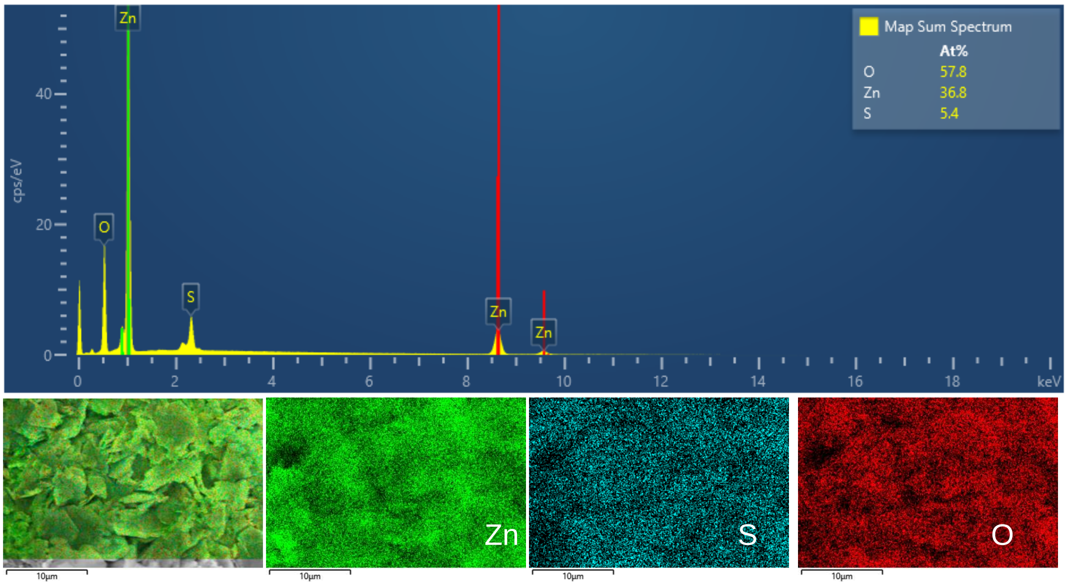

Figure S3. EDS mapping images of the cycled Zn anode in 1M $ZnSO_4$ (BE).

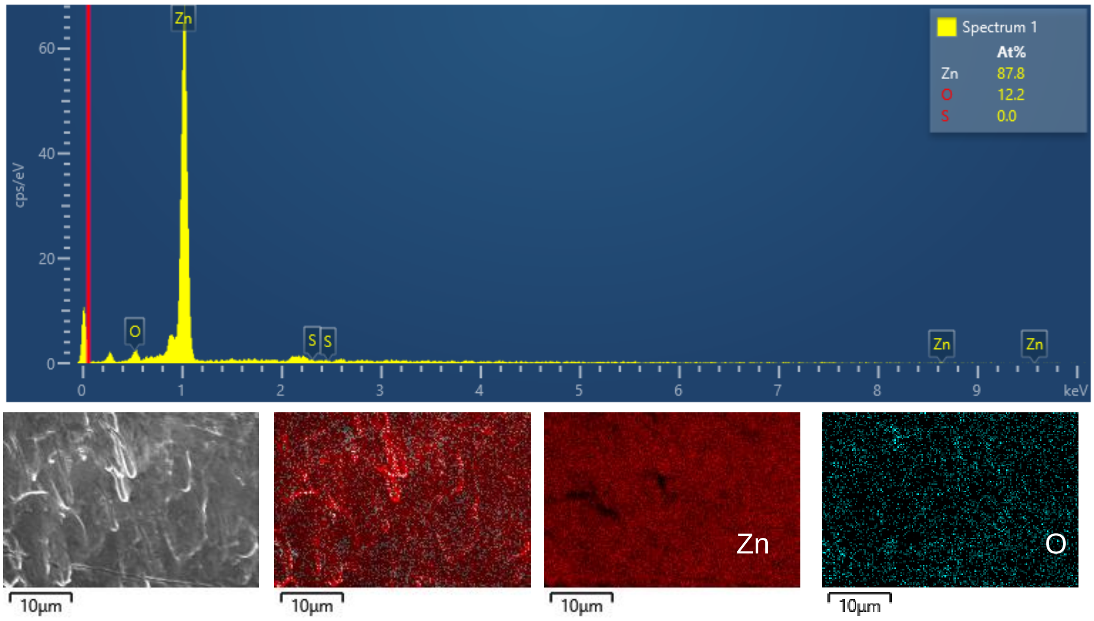

Figure S4. EDS mapping images of the cycled Zn anode in 1M $ZnSO_4$ + 25 mM NHP (DE).

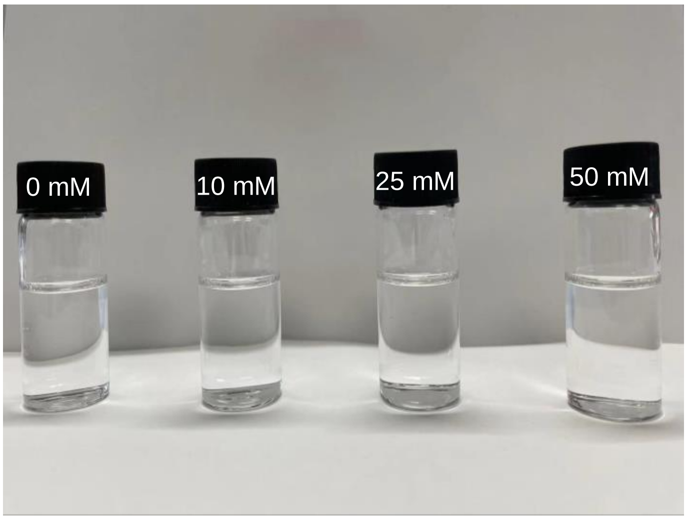

Figure S5. Digital photographs of the NHP containing electrolytes.

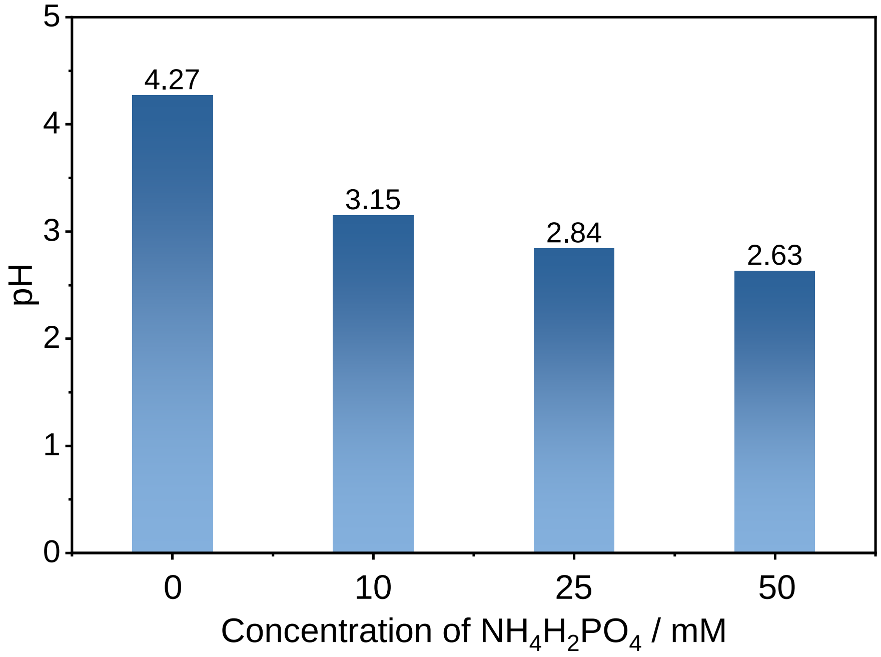

Figure S6. pH values of the NHP containing electrolytes.

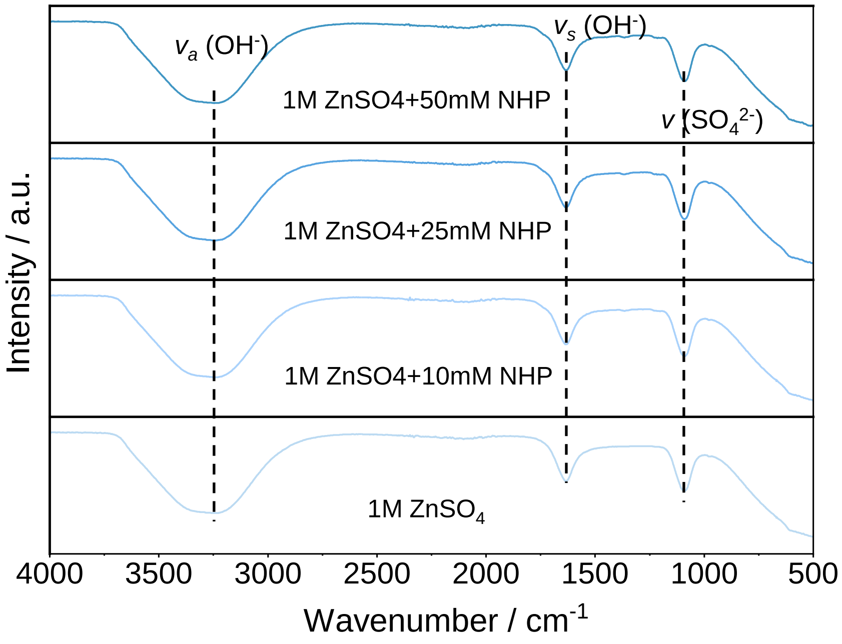

Figure S7. FT-IR spectra of electrolytes with different addition of NHP.

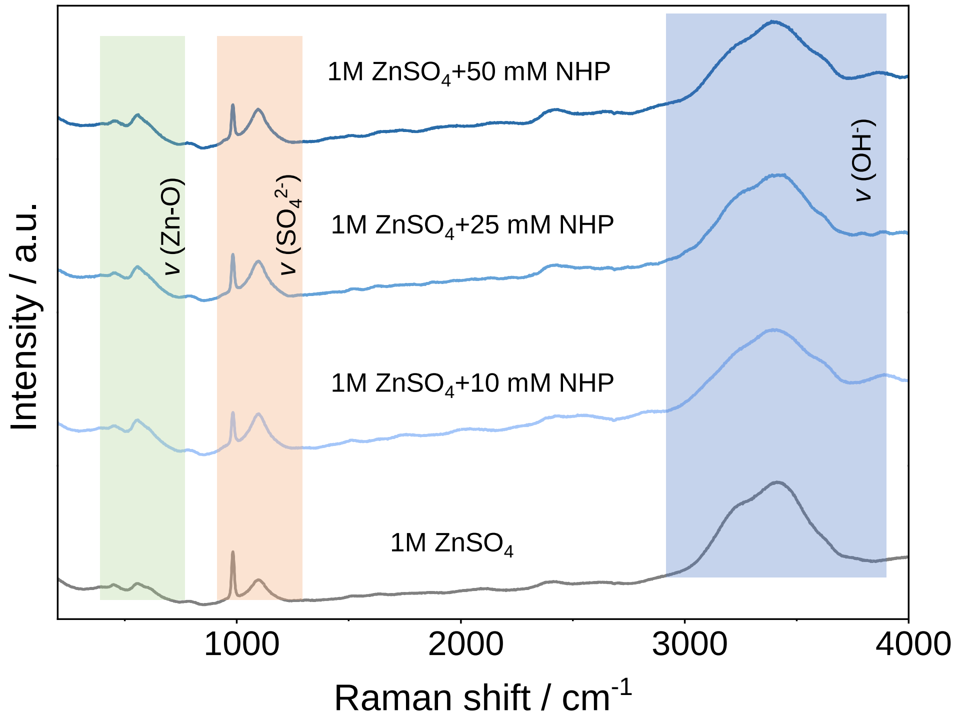

Figure S8. Raman spectra of electrolytes with different addition of NHP.

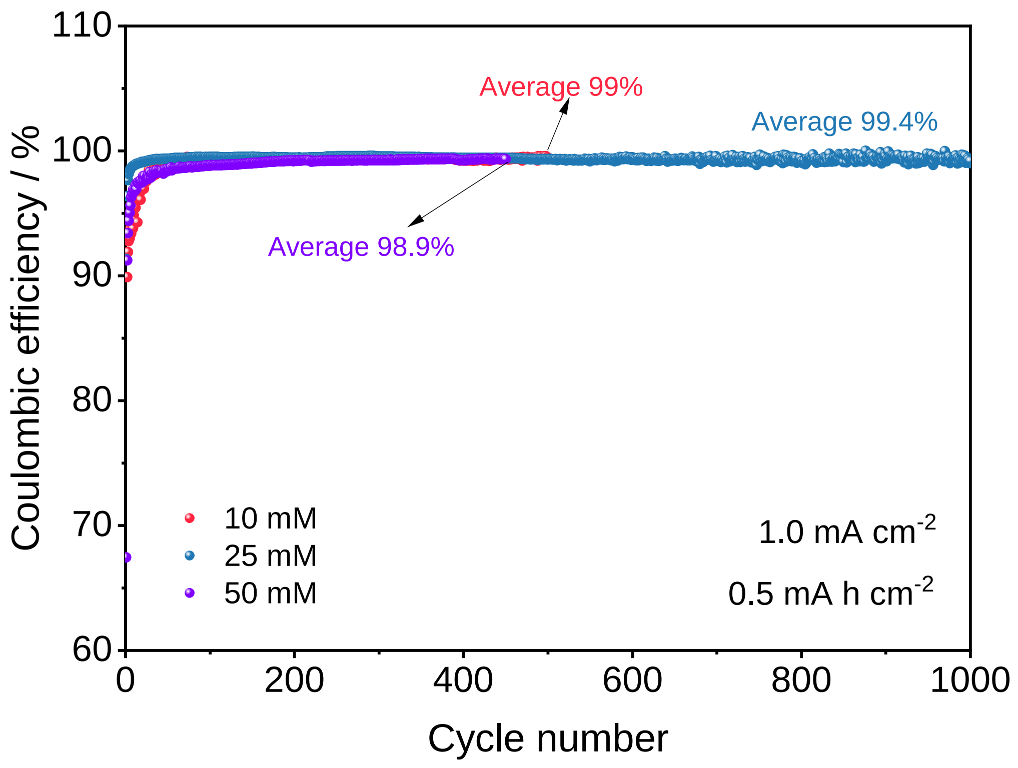

Figure S9. Coulombic efficiencies of Zn//Cu cells with different NHP containing electrolytes at 1 mA cm^{-2} with a fixed capacity of 0.5 mA h cm^{-2}.

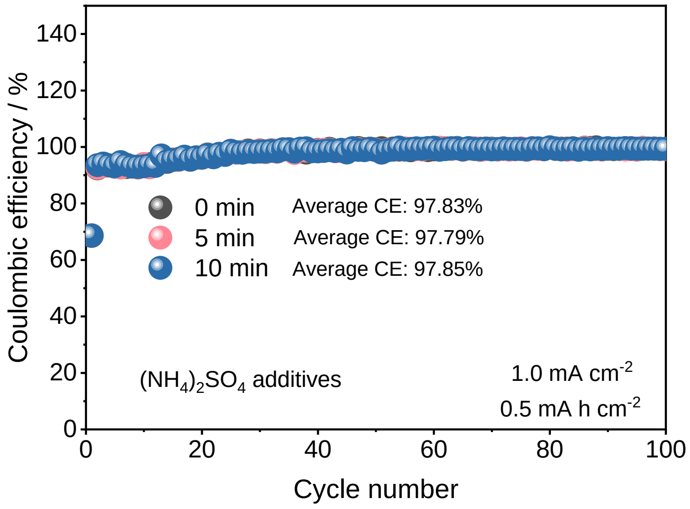

Figure S10. Coulombic efficiencies of Zn//Cu cells under the designed electrolyte with 25 mM NH_4^+ (12.5 mM $(NH_4)_2SO_4$) additive when different rest time was applied.

The Zn//Cu cells with $(NH_4)_2SO_4$ additive were subjected to the same condition of 1 mA cm^{-2} and 0.5 mA h cm^{-2}. Meanwhile, a rest period of 5 min or 10 min between the charge and discharge process was applied. As seen in Figure S10, all batteries displayed a similar initial CE and a similar average CE with ignorable deviations throughout 100 cycles. Therefore, there is no influence of the rest periods on the preferential NH_4^+ adsorption on the Zn surface, in line with the results in Figure 4a.

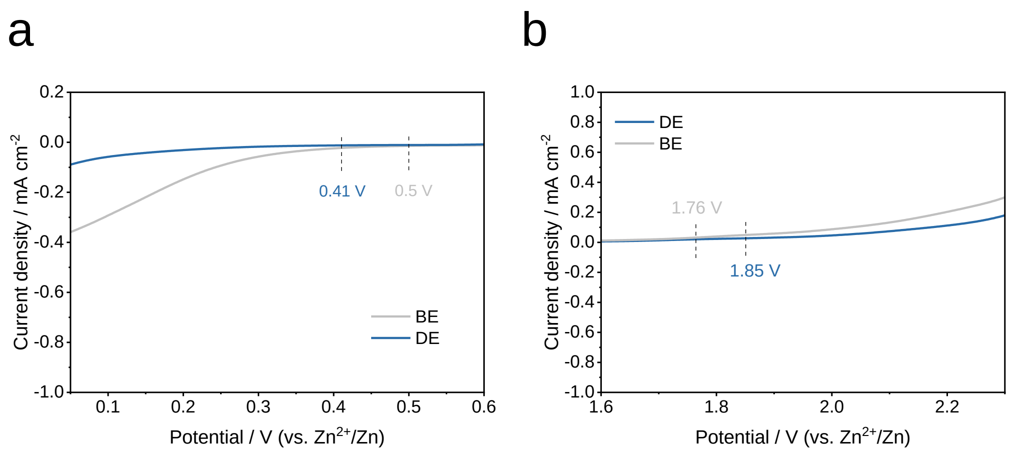

Figure S11. LSV curves presenting a) HER and b) OER in 1M $ZnSO_4$ (BE) and 1M $ZnSO_4$ + 25 mM NHP (DE).

Furthermore, the electrochemical stability windows of BE and DE were measured on the inactive Ti electrodes by linear sweep voltammetry (LSV) method. In Figure S11b, for the cathode side, the oxidation onset potential of DE increased from 1.76 V to 1.85 V (versus Zn^{2+}/Zn), indicating the oxygen evolution reaction (OER) was suppressed with addition of NHP. This can be ascribed to the lower pH value of DE (Figure S6), which corresponds to the results of the Pourbaix diagram of the $ZnSO_4$-H_2O system.[8] On the anode side (Figure S11a), similarly, the NHP additive drove the hydrogen evolution reaction (HER) onset potential from 0.5 V down to 0.41 V, manifesting DE undergoes a suppressed HER compared with BE. Therefore, an expanded electrochemical window of 1.44 V was obtained (Figure S11) with the presence of NHP.

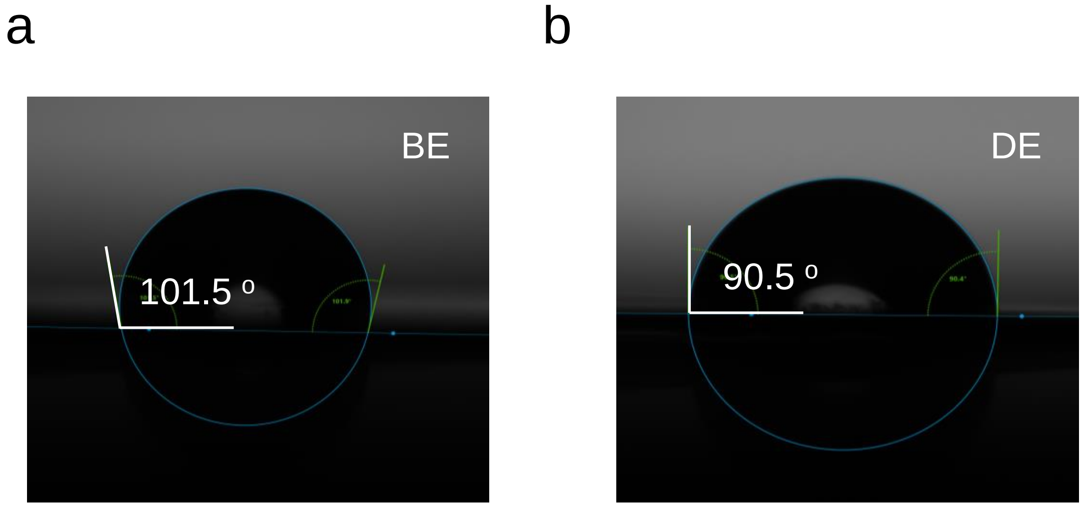

Figure S12. Contact angles of 1M $ZnSO_4$ (BE) and 1M $ZnSO_4$ + 25 mM NHP (DE) on the zinc foil surface.

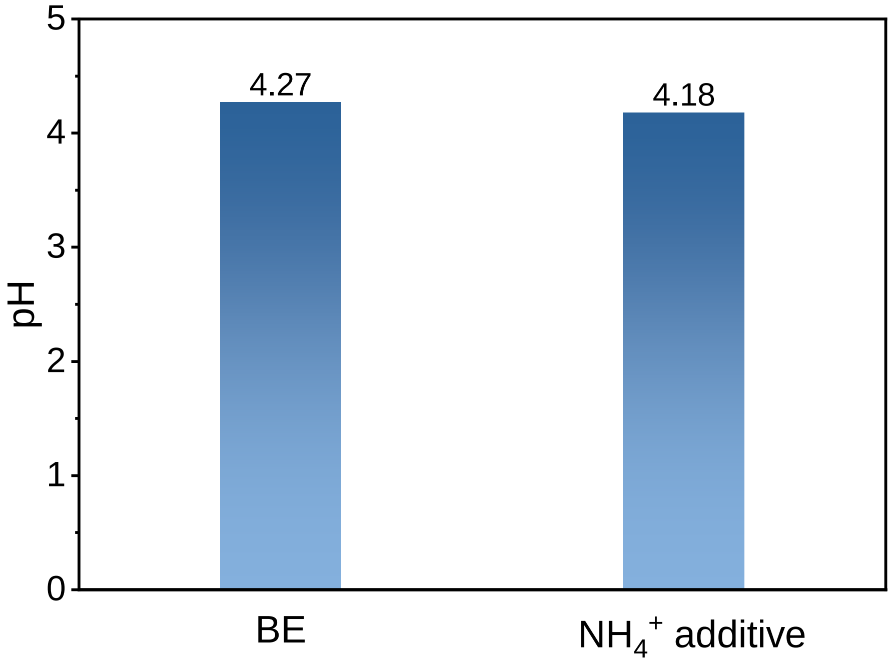

Figure S13. pH values of 1M $ZnSO_4$ (BE) and a designed electrolyte with 25 mM NH_4^+ (12.5 mM $(NH_4)_2SO_4$) additive.

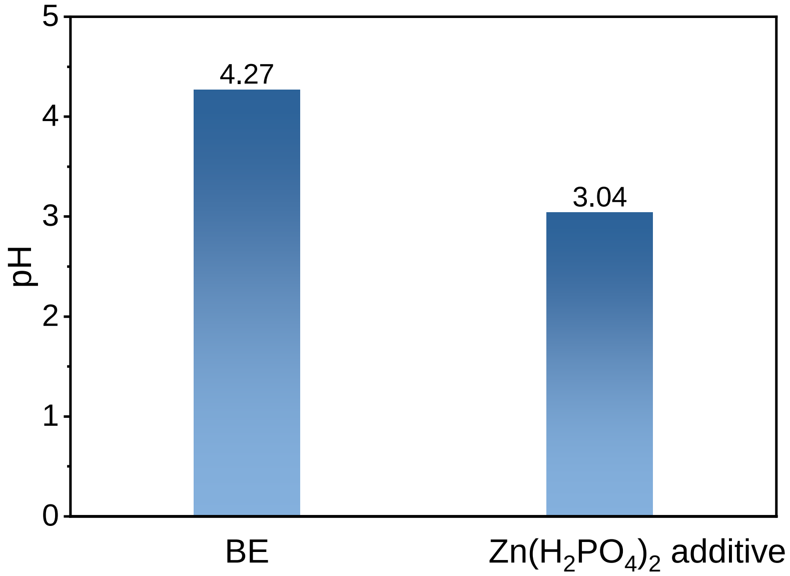

Figure S14. pH values of 1M $ZnSO_4$ (BE) and a designed electrolyte with 25 mM $(H_2PO_4)^-$ (12.5 mM $Zn(H_2PO_4)_2$) additive.

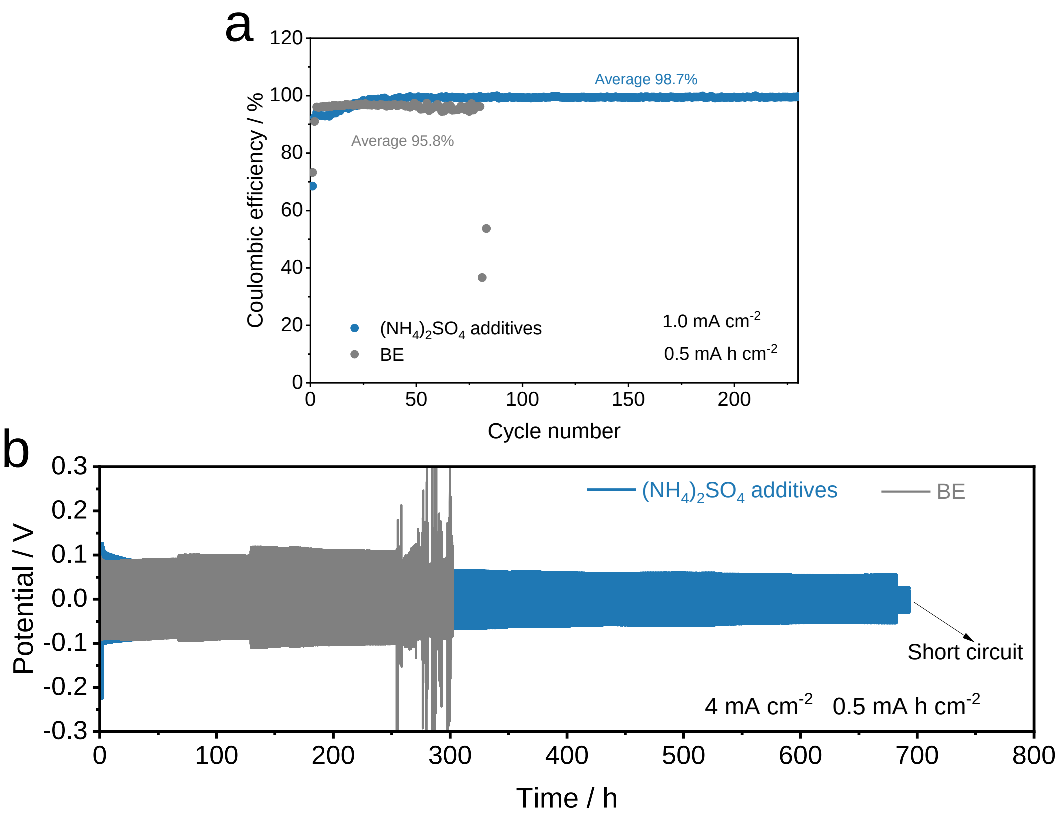

Figure S15. a) Coulombic efficiencies of Zn//Cu cells and b) cycling performances of Zn//Zn symmetric cells at 4 mA cm^{-2} with a capacity of 0.5 mA h cm^{-2} under 1M $ZnSO_4$ (BE) and the designed electrolyte with 25 mM NH_4^+ (12.5 mM $(NH_4)_2SO_4$) additive.

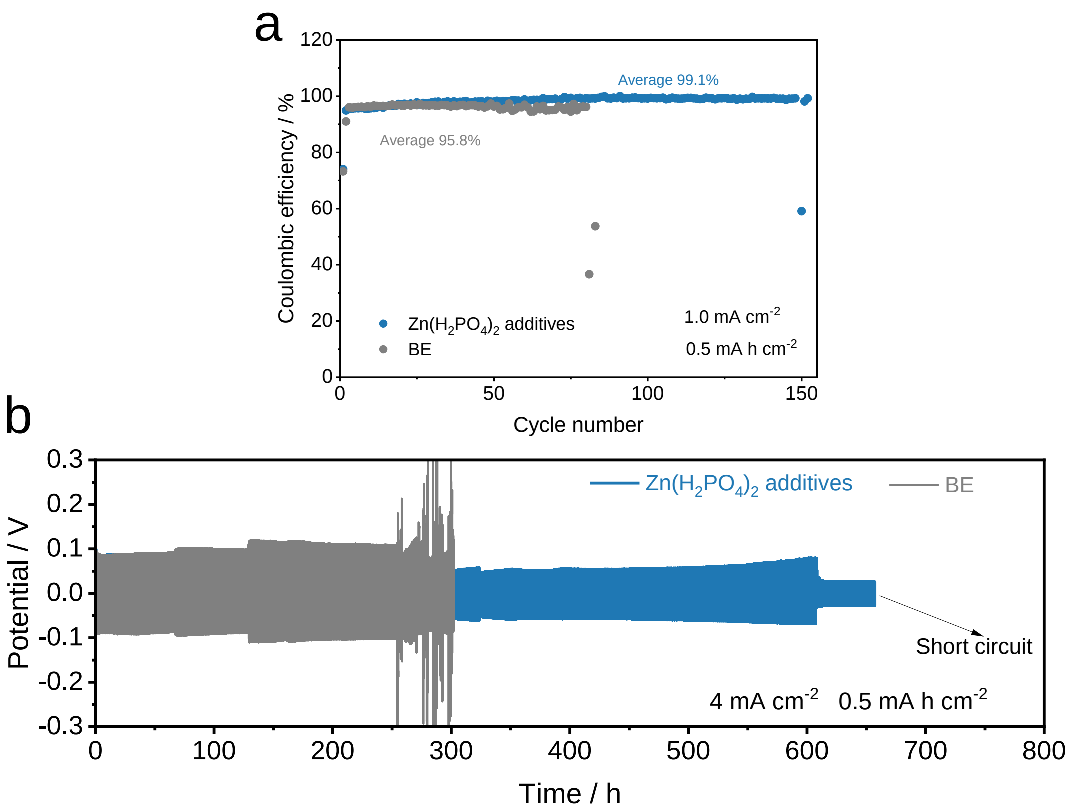

Figure S16. a) Coulombic efficiencies of Zn//Cu cells and b) cycling performances of Zn//Zn symmetric cells at 4 mA cm^{-2} with a capacity of 0.5 mA h cm^{-2} under 1 M $ZnSO_4$ (BE) and the designed electrolyte with 25 mM $(H_2PO_4)^-$ (12.5 mM $Zn(H_2PO_4)_2$) additive.

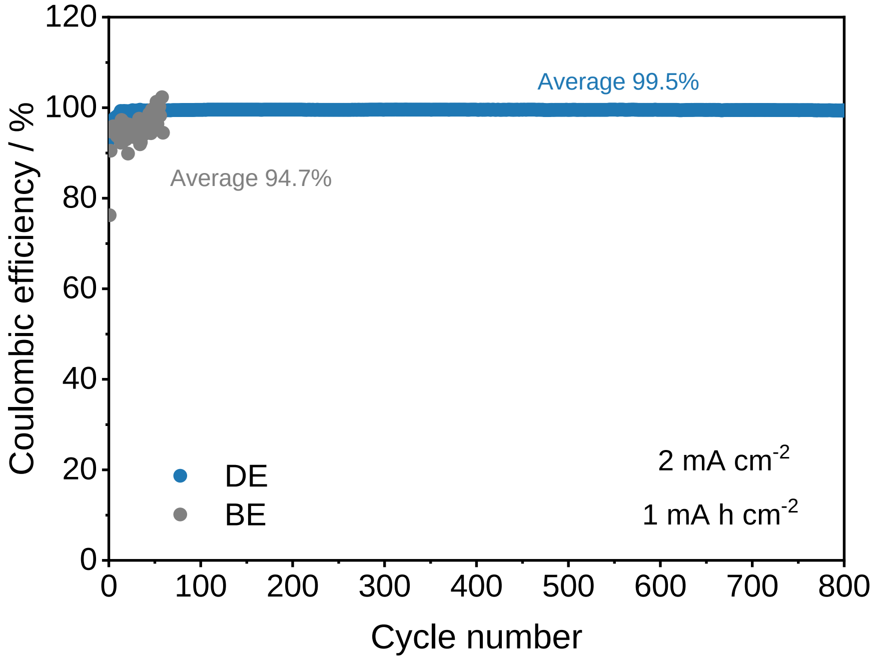

Figure S17. Coulombic efficiency of Zn//Cu asymmetric cells with/without NHP additives at 2 mA cm^{-2} and 1 mA h cm^{-2}.

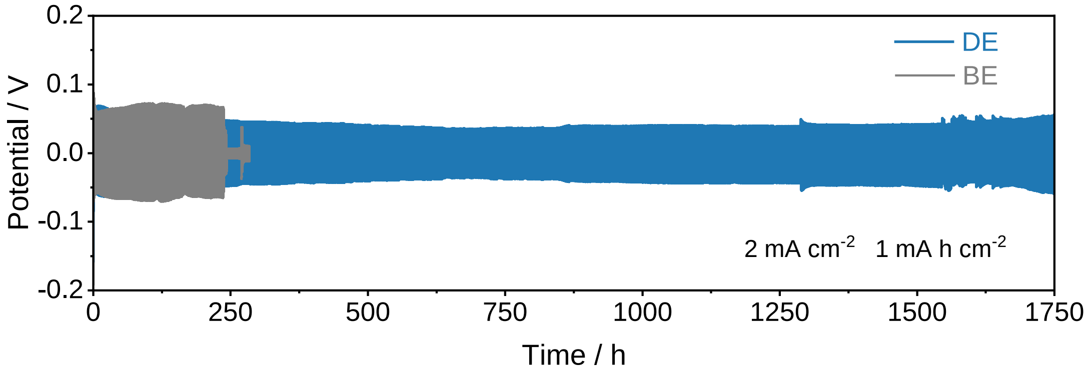

Figure S18. Plating/stripping cyclabilities of Zn//Zn symmetric cells under 1 M $ZnSO_4$ (BE) and 1 M $ZnSO_4$ + 25 mM NHP (DE) at 2 mA cm^{-2} with a capacity of 1 mA h cm^{-2}.

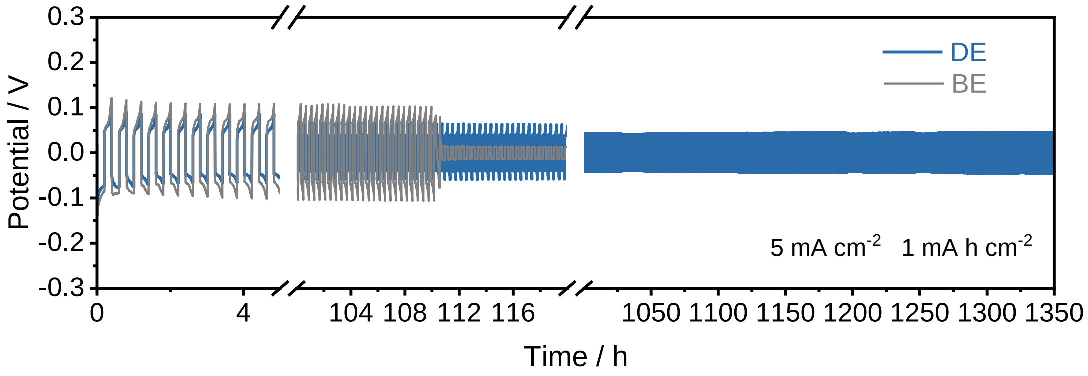

Figure S19. Plating/stripping cyclabilities of Zn//Zn symmetric cells under 1 M $ZnSO_4$ (BE) and 1 M $ZnSO_4$ + 25 mM NHP (DE) at 5 mA cm^{-2} with a capacity of 1 mA h cm^{-2}.

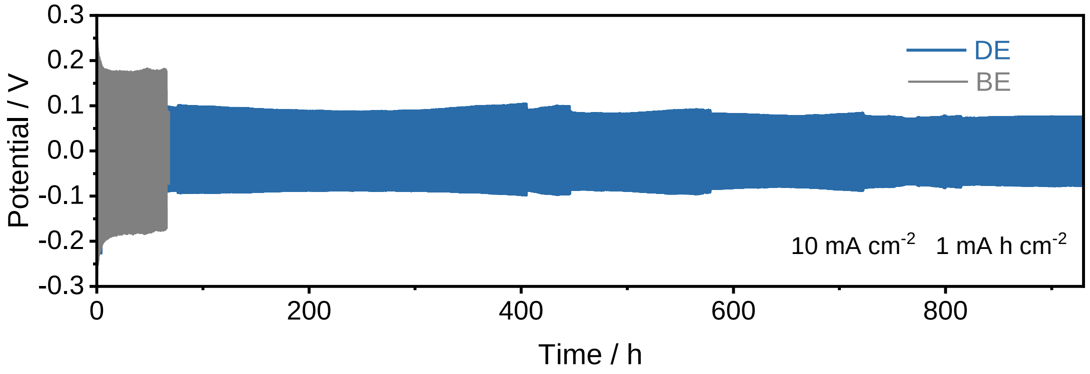

Figure S20. Plating/stripping cyclabilities of Zn//Zn symmetric cells under 1M $ZnSO_4$ (BE) and 1M $ZnSO_4$ + 25 mM NHP (DE) at 10 mA cm^{-2} with a capacity of 1 mA h cm^{-2}.

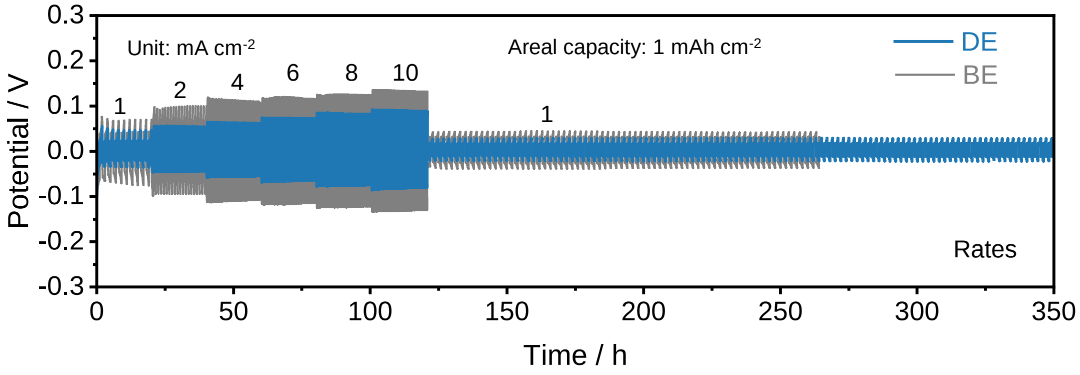

Figure S21. Plating/stripping cyclabilities of Zn//Zn symmetric cells under 1 M $ZnSO_4$ (BE) and 1 M $ZnSO_4$ + 25 mM NHP (DE) at different current densities with a fixed areal capacity of 1 mA h cm^{-2}.

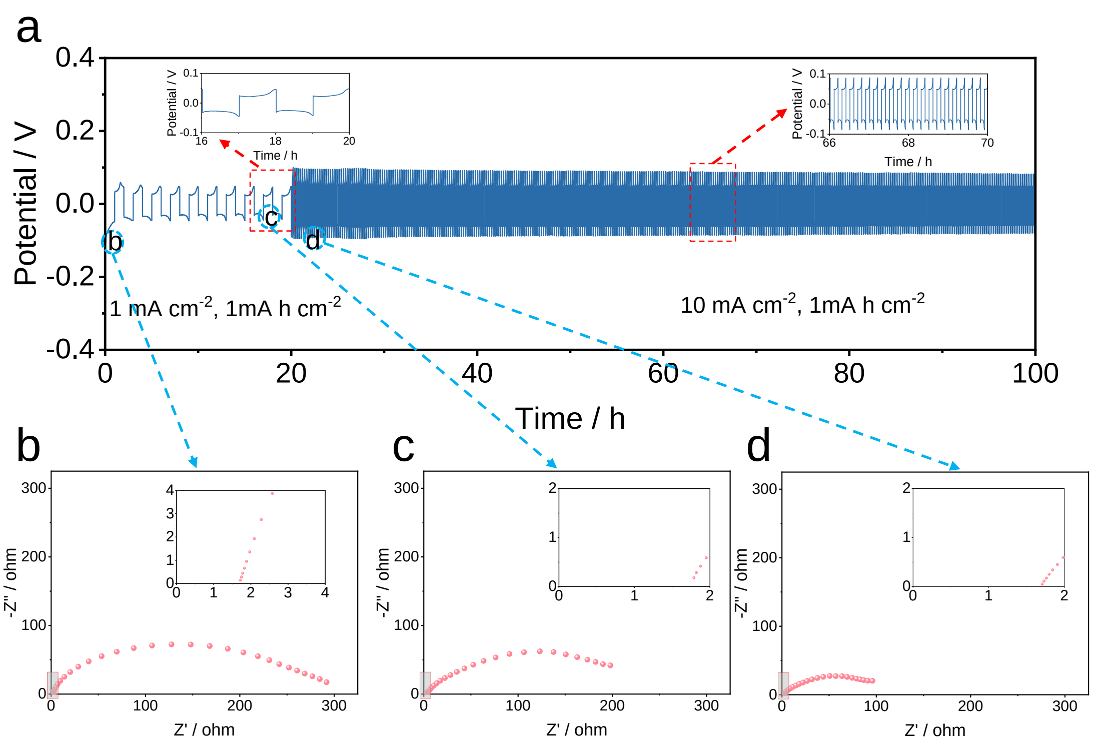

Figure S22. a) The voltage profile of pre-cycles (DE) at 1 mA cm^{-2} and 1 mA h cm^{-2} followed by 10 mA cm^{-2} and 1 mA h cm^{-2}, insets show the amplified profile at different cycles. The corresponding impedance spectra of the positions in a): b) before test, c) after 18 h at 1 mA cm^{-2} and 1 mA h cm^{-2}, d) after 3 h at 10 mA cm^{-2} and 1 mA h cm^{-2}.

From Figure S22, an overpotential of ~60 mV could be seen for a symmetric Zn cell at 1 mA cm^{-2}. This value increases to 100 mV at 10 mA cm^{-2}. Figure S22b and S22c demonstrated an obvious decline of R_{ct} when the battery was subjected to 1 mA cm^{-2} and 1 mA h cm^{-2}. Then the value declines to a moderate value under 10 mA cm^{-2} (Figure S22d). According to the Zhi's protocol,[9] there is no sign of short circuit nor an abrupt R_{ct} drop throughout the whole process, which confirms the good cycling stability of the Zn//Zn cell with DE.

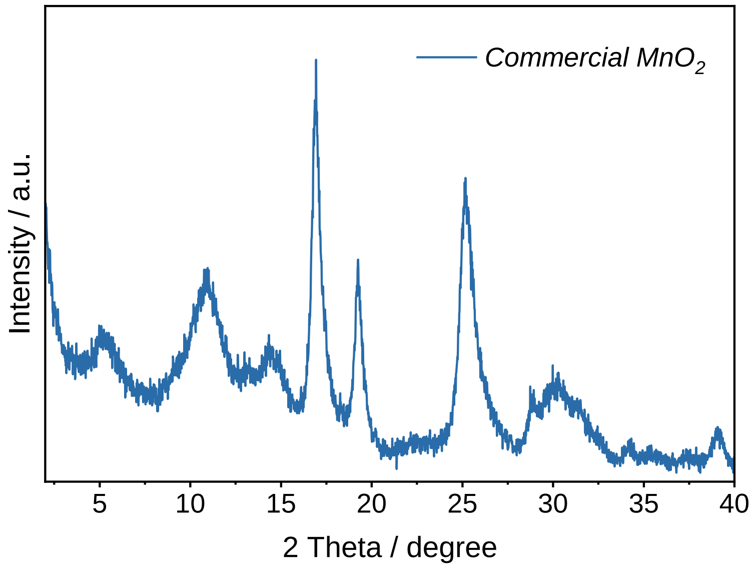

Figure S23. XRD pattern (Mo K_{α} radiation) of the commercial MnO_2 powder.

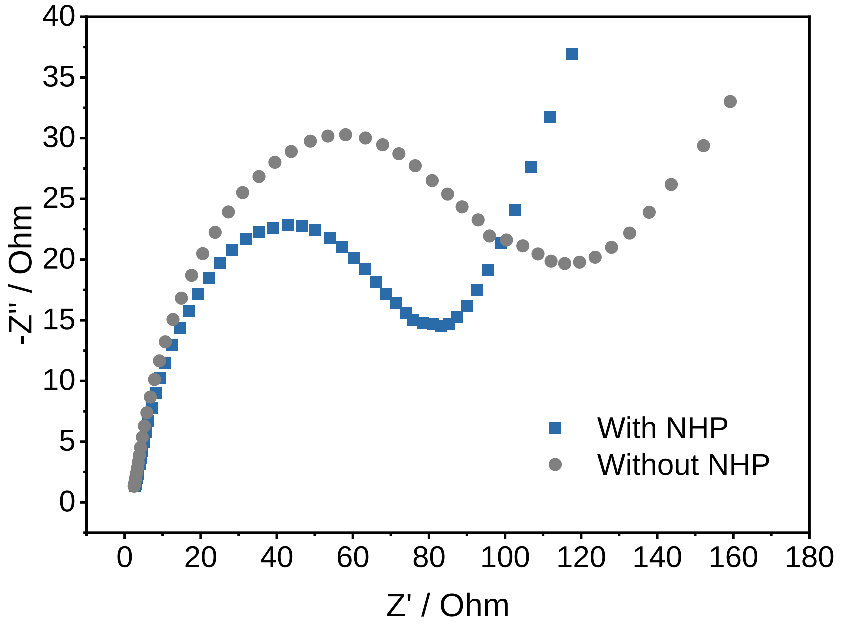

Figure S24. EIS spectra of Zn//MnO_2 full cells with/without NHP.

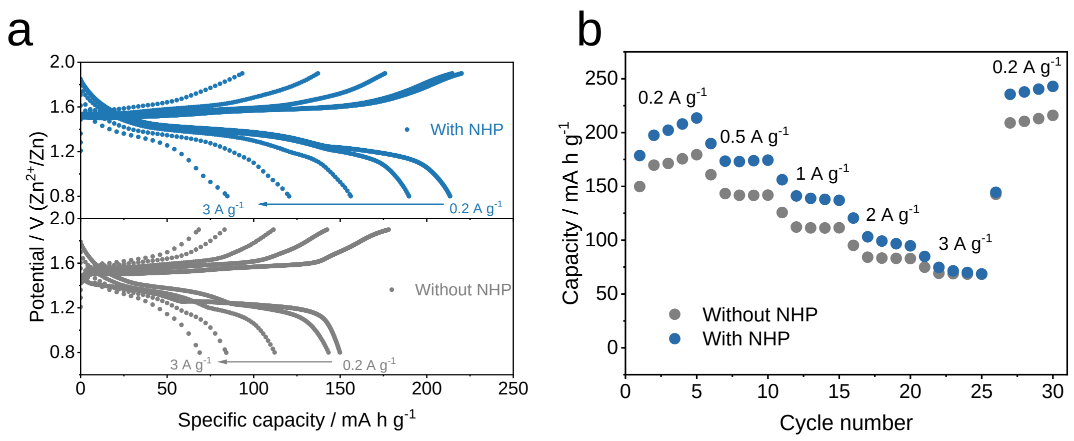

Figure S25. a) Charge/discharge curves and b) rate performances of Zn//MnO_2 cells in a large N/P ratio.

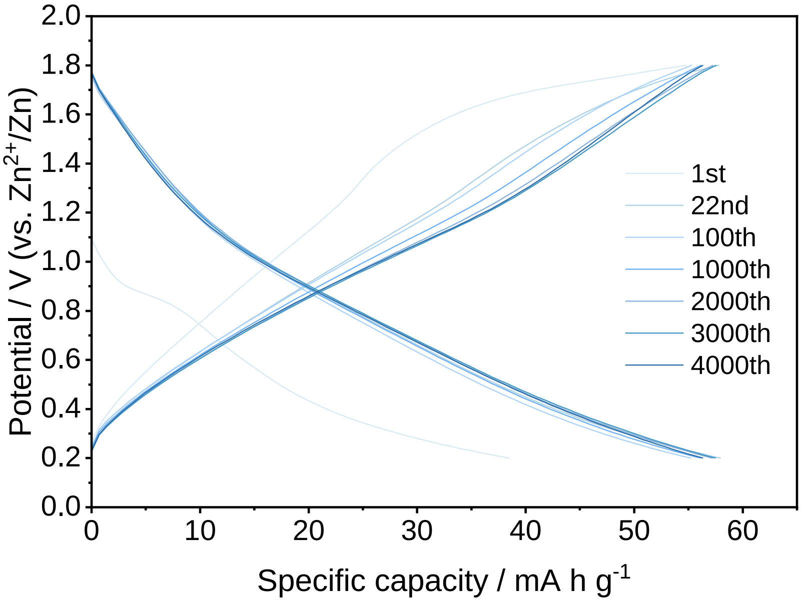

Figure S26. Charge-discharge curves of the Zn//AC capacitor under 1M $ZnSO_4$ + 25 mM NHP (DE) at 0.5 A g^{-1} in different cycles.

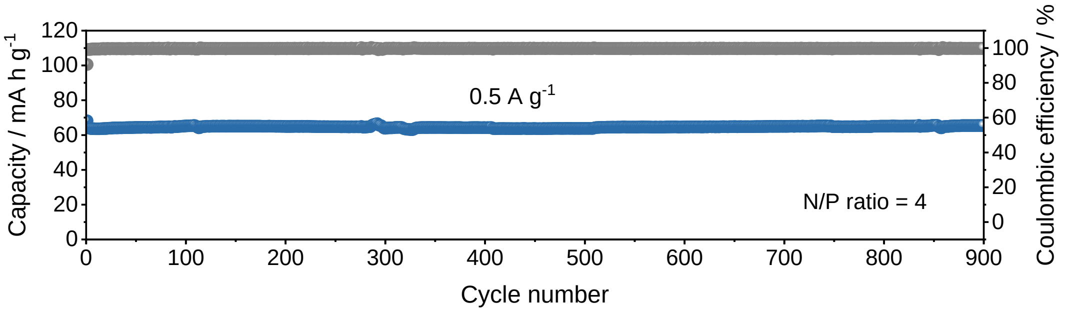

Figure S27. Cycling performance of the Zn//AC capacitor with NHP at 0.5 A g^{-1} when a low N/P ratio of 4 was adopted.

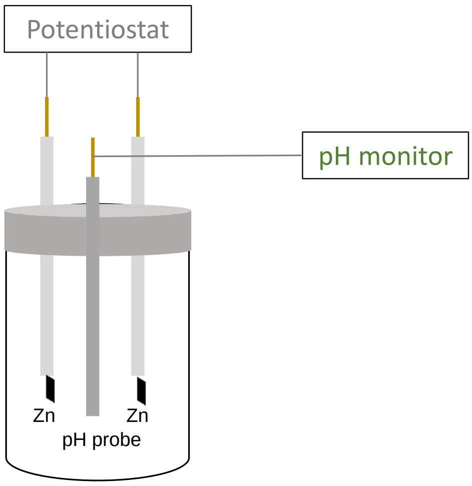

Figure S28. The designed system for monitoring pH values of electrolytes upon battery cycling.

The *in-situ* pH measurements were conducted on a home-designed configuration as displayed in Figure S28 using an electrolytic cell (50 mL) with 15 mL electrolyte. Two pieces of Zn foils (1×1 cm; 100 μm in thickness) with a distance of 1~2 cm were connected with the potentiostat; meanwhile, a pH probe was combined with these two Zn foils to construct the pH Zn//Zn symmetric cell. This cell was subjected to 10 mA cm^{-2} by the potentiostat. Therefore, pH values were recorded by the pH monitor during battery cycling.

Table S1. The comparison of this work with other previous aqueous electrolytes for zinc metal anodes.

Electrolyte (M: mol L^{-1}; m: mol kg^{-1})	Symmetric Zn cells: Current Density ($mA\ cm^{-2}$)/ Capacity ($mAh\ cm^{-2}$)	Symmetric Zn cells: Lifespan (hours)	Asymmetric Zn cells: Current Density ($mA\ cm^{-2}$)/ Capacity ($mAh\ cm^{-2}$)	Asymmetric Zn cells: Average CE	Asymmetric Zn cells: Lifespan (cycles)	Ref.
1 M $ZnSO_4$+ 25 mM NHP	1/1<br>4/0.5<br>5/5	2100<br>1900<br>315	1/0.5	99.4%	1000	This work
30 m $ZnCl_2$	0.2/0.033	600	1/0.4	95.4%	95	[10]
2 M $ZnSO_4$+0.05 mM TBA_2SO_4	2/2<br>5/5	300<br>160	10/10	98%	1	[11]
1.3 m $ZnCl_2$/H_2O-DMSO (H_2O/DMSO = 4.3:1 by volume)	0.5/0.5	1000	1/0.5	99.5%	400	[12]
3 m $ZnSO_4$+2 m LiCl	1/1	125	N.A.	N.A.	N.A.	[8]
2 M $ZnSO_4$+0.1 M BIS-TRIS	1/1	1200	1/1	98.5%	400	[13]
2 M $ZnSO_4$+0.05 mg mL^{-1} $Ti_3C_2T_x$ MXene	1/1<br>4/1	1000<br>250	1/1	98.32%	120	[14]
1 M $ZnSO_4$+10 mM glucose	2/2	700	1/0.5	97.2%	230	[15]
2 M $ZnSO_4$+0.5 g L^{-1} TMBAC	1/2	1000	1/1	99%	200	[16]
5 m $ZnCl_2$+5 m Betaine	0.5/0.5	1400	0.5/0.5	98%	400	[17]
1 M $ZnSO_4$+5 mM Thiourea	1/1	1200	10/1	98.9%	700	[18]
1 M $Zn(ClO_4)_2$+10 mM β-cyclodextrin	1/1	1000	N.A.	97.6%	530	[19]
2 M $ZnSO_4$+ 10 mM Cysteine	0.5/0.5	2300	5/5	99.4%	280	[20]
2 M $ZnSO_4$+ 10 vol.% DMA	1/5	250	2/1	98.7	250	[21]

*Note: Tetrabutylammonium sulfate (TBA_2SO_4); Dimethyl sulfoxide (DMSO); 2Bis(2-hydroxyethyl) amino-2-(hydroxymethyl)-1,3-propanediol (BIS-TRIS); Benzyltrimethylammonium chloride (TMBAC); *N,N*-Dimethylacetamide (DMA)

References

[1] H. Dong, J. Li, S. Zhao, Y. Jiao, J. Chen, Y. Tan, D. J. L. Brett, G. He, I. P. Parkin, *ACS Appl. Mater. Interfaces* **2021**, *13*, 745-754.

[2] C. Huang, X. Zhao, S. Liu, Y. Hao, Q. Tang, A. Hu, Z. Liu, X. Chen, *Adv. Mater.* **2021**, *33*, 2100445.

[3] W. Kohn, L. J. Sham, *Phys. Rev.* **1965**, *140*, A1133-A1138.

[4] P. Giannozzi, S. Baroni, N. Bonini, M. Calandra, R. Car, C. Cavazzoni, D. Ceresoli, G. L. Chiarotti, M. Cococcioni, I. Dabo, A. Dal Corso, S. de Gironcoli, S. Fabris, G. Fratesi, R. Gebauer, U. Gerstmann, C. Gougoussis, A. Kokalj, M. Lazzeri, L. Martin-Samos, N. Marzari, F. Mauri, R. Mazzarello, S. Paolini, A. Pasquarello, L. Paulatto, C. Sbraccia, S. Scandolo, G. Sclauzero, A. P. Seitsonen, A. Smogunov, P. Umari, R. M. Wentzcovitch, *J. Phys. Condens. Matter* **2009**, *21*, 395502.

[5] H. J. Monkhorst, J. D. Pack, *Phy. Rev. B* **1976**, *13*, 5188-5192.

[6] S. A. Grimme, J.; Ehrlich, S.; Krieg, H. A, *J. Chem. Phys.* **2010**, *132*, 154104.

[7] Z. Xu, X. Lv, J. Chen, L. Jiang, Y. Lai, J. Li, *Carbon* **2016**, *107*, 885-894.

[8] X. Guo, Z. Zhang, J. Li, N. Luo, G.-L. Chai, T. S. Miller, F. Lai, P. Shearing, D. J. L. Brett, D. Han, Z. Weng, G. He, I. P. Parkin, *ACS Energy Lett.* **2021**, *6*, 395-403.

[9] Q. Li, A. Chen, D. Wang, Z. Pei, C. Zhi, *Joule* **2022**, *6*, 273-279.

[10] C. Zhang, J. Holoubek, X. Wu, A. Daniyar, L. Zhu, C. Chen, D. P. Leonard, I. A. Rodriguez-Perez, J. X. Jiang, C. Fang, X. Ji, *Chem. Commun.* **2018**, *54*, 14097-14099.

[11] A. Bayaguud, X. Luo, Y. Fu, C. Zhu, *ACS Energy Lett.* **2020**, *5*, 3012-3020.

[12] L. Cao, D. Li, E. Hu, J. Xu, T. Deng, L. Ma, Y. Wang, X. Q. Yang, C. Wang, *J. Am. Chem. Soc.* **2020**, *142*, 21404-21409.

[13] M. Luo, C. Wang, H. Lu, Y. Lu, B. B. Xu, W. Sun, H. Pan, M. Yan, Y. Jiang, *Energy Storage Mater.* **2021**, *41*, 515-521.

[14] C. Sun, C. Wu, X. Gu, C. Wang, Q. Wang, *Nano-Micro Lett.* **2021**, *13*, 89.

[15] P. Sun, L. Ma, W. Zhou, M. Qiu, Z. Wang, D. Chao, W. Mai, *Angew. Chem. Int. Ed.* **2021**, *60*, 18247-18255.

[16] K. Guan, L. Tao, R. Yang, H. Zhang, N. Wang, H. Wan, J. Cui, J. Zhang, H. Wang, H. Wang, *Adv. Energy Mater.* **2022**, *12*, 2103557.

[17] Z. Jia, W. Zhao, S. Hu, X. Yang, T. He, X. Sun, *Chem. Commun.* **2022**, *58*, 8504-8507.

[18] H. Qin, W. Kuang, N. Hu, X. Zhong, D. Huang, F. Shen, Z. Wei, Y. Huang, J. Xu, H. He, *Adv. Funct. Mater.* **2022**, 2206695.

[19] M. Qiu, P. Sun, Y. Wang, L. Ma, C. Zhi, W. Mai, *Angew. Chem. Int. Ed.* **2022**, *61*, e202210979.

[20] Q. Meng, R. Zhao, P. Cao, Q. Bai, J. Tang, G. Liu, X. Zhou, J. Yang, *Chem. Eng.*

J. **2022**, *447*, 137471.
[21] F. Wu, Y. Chen, Y. Chen, R. Yin, Y. Feng, D. Zheng, X. Xu, W. Shi, W. Liu, X. Cao, *Small* **2022**, *18*, 2202363.
